# An inclusive parametric study for performance improvement in WEDM process of pure titanium using Naive Bayes classifier

**DOI:** 10.1038/s41598-025-19360-5

**Published:** 2025-10-09

**Authors:** Jay Vora, Shahil Rathod, Masoud Seidi, Saeed Yaghoubi, Rakesh Chaudhari, Subraya Krishna Bhat

**Affiliations:** 1 Department of Mechanical Engineering, School of Technology, Pandit Deendayal Energy University, Gandhinagar, Gujarat 382007 India; 2 Department of Computer Engineering, Faculty of Engineering, Ilam University, Ilam, Iran; 3 Department of Mechanical Engineering, Faculty of Engineering, Ilam University, Ilam, Iran; 4 Department of Mechanical and Industrial Engineering, Manipal Institute of Technology, Manipal Academy of Higher Education, Manipal, Karnataka 576104 India

**Keywords:** Design of experiment, Taguchi design, Box–Behnken design, Wire-electrical discharge machining, Naive Bayes, Materials science, Engineering, Mechanical engineering

## Abstract

Titanium alloys have exceptional hardness and high toughness, which can cause significant challenges in traditional machining. Wire-electrical discharge machining (WEDM) process offers excellent accuracy and high precision compared to conventional machines. Design of experimental (DOE) technique provides a systematic way to conduct the experimental runs with the least trials by saving time and cost. Thus, the current work focuses on the modelling of WEDM process at numerous input process environments using Taguchi and BBD-RSM approach. The variable input factors of WEDM process include pulse-on-time (T_on_), pulse current (I_p_), and pulse-off-time (T_off_), whereas the response measures of material removal rate (MRR) and surface roughness (SR) were taken. The performance and adequacy of Taguchi and BBD-RSM models were assessed by using ANOVA, coefficient of determination (R^2^), and residual plots. The effect of WEDM factors on performance measures was studied by using main effect plots. Based on Entropy criterion, the weights of MRR and SR response factors were computed to, in turn, 0.52 and 0.48. The practical tests defined in the DOE along with the MRR and SR were considered as inputs to the Naive Bayes (NB) predictive model. The prediction findings indicated the appropriate performance of the NB algorithm. The authors believe that the present study, which compares DOE techniques and their application in predicting process outcomes using Naive Bayes classifier, will be useful for users in different domains and various applications.

## Introduction

Titanium is a highly versatile metal with exceptional corrosion resistance, impressive strength-to-weight ratio, fatigue resistance, and also remains stable at high temperature^1,2^. Owing to their exceptional properties, titanium has become an important material in industry, providing reliability and durability in various applications^3,4^. Titanium and its alloys are widely used in many industries such as chemical sector, aerospace engineering, food industries, biomedical applications and automotive manufacturing^5^. Due to the excellent biocompatibility, they are widely used in biomedical fields, and suitable joints for bone, staple pins, dental implants, surgical instruments, and other body tissues^6,7^. Corrosion resistance property ensures the durability with human body, withstand physiological stresses, and compatibility with healing and stability of implants^8^. Titanium alloys have exceptional hardness and high toughness, which can cause significant challenges in traditional machining such as higher tool wear, rough surface finish, and more machining forces etc.^9,10^. Wire-electrical discharge machining (WEDM) process offers excellent accuracy and high precision compared to conventional machines^11^. WEDM of advanced materials including titanium and its alloys is capable of producing complex and intricate geometries^12,13^. EDM is used for various applications such as die manufacturing, precision dies, medical industries, aerospace and automobile sectors^14,15^. A Series of electric sparks is generated between a workpiece and wire electrode, which removes the material from the work surface^16,17^. Pulse of electric energy passes through, causing localised vaporisation of material from the workpiece. A continuous stream of dielectric fluid flows in the machining zone, maintaining spark stability, better cooling effect, and debris removal^18^. It is necessary to have both work and tool materials to be electrically conductive^19^. As WEDM process consists of numerous control variables and response measures, precise controlling of these factors becomes essential to acquire desired outcomes^20^. WEDM consists of input factors of pulse-on-duration (T_on_), duty cycle, pulse current (I_p_), and pulse-off-duration (T_off_), wire tension, wire feed rate, etc. During the precise machining of hard materials like titanium alloys, higher material removal rate (MRR) for increased productivity, and lower surface roughness (SR) for smooth surface finish are always desired output measures. To understand the effect of multiple input factors, a strategic planning of experimentation is required. Design of experimental (DOE) technique provides a systematic way to conduct the experimental runs with the least trials by saving time and cost^21–23^. Taguchi, and Box-Behnken design (BBD) of response surface methodology (RSM) are the most popular DOE techniques.

### Taguchi design approach

Genichi Taguchi developed the Taguchi DOE approach to enhance process robustness and optimise product quality^24^. It consists of system design, which identifies the levels of input factors, parameter design, which identifies the optimal level of input factors to attain best performance, and tolerance design, which impacts the quality by fine-tuning the factors by adjusting the tolerance levels^25,26^. In comparison with full factorial design, Taguchi’s design needs fewer experimental trials, which in turn enhances the design efficacy. Orthogonal array (OA), and signal-to-noise (S/N) ratio are the two main attributes of Taguchi design^27^. Use of OA provides smaller experimental trials, which saves time, and charges required for experimentation^28^. On the other hand, S/N ratio is useful to determine the deviance of performance measures from anticipated levels. This includes, lower-the-better, nominal-the-better, and higher-the-better criterias. Taguchi’s approach provides a linear interrelationship among the input factors and output measures as shown in Eq. (1).

1 $$y= {\beta }_{0}+ {\beta }_{1}{X}_{1}+ {\beta }_{2}{X}_{2}+ .. .+ {\beta }_{K}{X}_{A}+ \alpha$$

where A represent the number of factors, $\alpha$ represents the error term.

### BBD-RSM design approach

RSM design approach was introduced by Box and Wilson in a year 1950s, which gives fairly good empirical performance^29^. RSM is a group of statistical and mathematical methods used for modelling and analysing the system performance by giving an interrelationship among the input factors and output measures^30,31^. Such applications are of great importance and typically influenced by numerous variables. The goal of engineering applications is to identify the variables that can improve this response. RSM is extensively functional across various industrial settings and parameter optimisation processes, including electronic manufacturing, semiconductor, metal cutting operations, electronic manufacturing and chemical^32^. BBD design of RSM approach is highly effective while considering the three-factor experimental design, which requires less number of trials, which still gives second-order interactions with nonlinear effects. BBD does not include extreme conditions of factors, which in turn eliminates the impractical parameter settings. It produces a non-linear second-order polynomial relationship as shown in Eq. (2):

2 $$y= {\beta }_{0}+ {\sum }_{i=1}^{A}{\beta }_{i}{X}_{i}+ {\sum }_{i=1}^{A}{\beta }_{ii}{X}_{i}^{2}+ \sum \sum_{i<j}{\beta }_{ij}{X}_{i}{X}_{j}+ \alpha$$

Least squares method is used in the RSM to obtain the coefficients of the functions.

Multiple studies were reported on the use of Taguchi and RSM design individually for analysis of EDM performance. Jagdale et al.^33^ used Taguchi’s L9 OA for WEDM Ti6Al4V alloy. The key input parameters examined include I_p_, T_on_, and T_off,_ each tested at three stages with MRR, and SR were measured as output responses. The findings highlighted that I_p_, T_on_ were the most influential factors affecting MRR and SR, contributing 72.75% and 11.68%, respectively, to the variations in results. The study emphasised that optimising peak current and T_on_ was crucial for optimize machining performance in WEDM of Ti6Al4V alloy. Hoang-Vuong et al.^34^ used a Taguchi-TOPSIS method in optimising EDM parameters of titanium alloy. The study successfully identified the optimal process parameters for EDM using a graphene-coated electrode. The best configuration U of 55 V, I_p_ of 5 A, and T_on_ of 1500 µs achieves a MRR of 6.57 mg/min. Singh and Kirkup^35^ employed Taguchi’s methodology to enhance the WEDM process parameters for increasing MRR of Titanium Grade 5 alloy. Experimental results were converted into Signal-to-Noise (S/N) ratio values, and a main effect plot was generated. ANOVA identified primary control parameters, with T_on_ having the major impact, followed by T_off_ and gap voltage. A confirmation test validated the findings, showing a substantial 13.5% improvement in the S/N ratio at the optimised condition compared to the initial setup. Chaudhari et al.^36^ investigated the performance of powder-mixed EDM of Udimet 720 using Taguchi’s L9 design. The developed empirical equations have successfully obtained optimised parameters for the elected output responses. Statistical analysis performed by ANOVA, coefficient of determination confirmed the suitability of Taguchi design. The validated trials were found to be within an acceptable variation range.

Selverajan et al.^37^ used RSM design during EDM of Si3N4-based ceramic composites. Their findings have revealed a significant improvement in the performance metrics, including MRR, EWR and geometrical tolerances. The optimal EDM parameters consist of current at 8 A, T_on_ at 20 µs, and T_off_ at 5 µs, and dielectric pressure of 28 kg/cm^2^. At high pulse current, increased plasma energy improved the circularity of the machined hole. The study reported by Kumar et al.^38^ carried out a parametric study using BBD approach of RSM to enhance the machining performance of EDM process for Ti6Al4V alloy. Using RSM DOE approach, the performance of input factors of I_p_, discharge voltage, and T_on_ was investigated on MRR and SR measures. The obtained results have shown a higher impact of found that I_p_ and T_on_ on both output measures. The optimal input parameters of I_p_ at 15A, voltage of 50 V, and T_on_ of 150 µs have shown improved surface conditions and higher erosion rate. Mahanti and Das^39^ used BBD design of RSM for modelling and optimisation of EDM process using ML technique. The combined approach of RSM design with ML technique has shown improved performance of H13 alloy. Chaudhari et al.^40^ attained multiple output measures of WEDM process parameters of WEDM using a combined approach of RSM and passing vehicle search algorithm. The past studies conducted by Rajkumar et al.^41^ also preferred RSM design along with optimisation technique to enhance performance characteristics of EDM process for Inconel 718. Their obtained results have shown the suitability of the DOE technique along with an integrated approach of optimisation method.

In data science, there is a need to employ various tools for categorising data, one of the most important of which is the Naive Bayes (NB) algorithm. In machine learning, a Naive Bayes classifier consists of straightforward probability-based classifiers built upon Bayes’ theorem, assuming that random variables are independent^42–44^. Essentially, the Naive Bayes method allows for classifying phenomena by determining the probability of an event occurring or not^45^. This technique is one of the simplest predictive algorithms, known for its satisfactory accuracy. Machine learning has a wide range of applications in engineering problems, helping to improve performance, increase efficiency, and reduce costs^46,47^.

Thus, the studied literature has suggested that the use of DOE technique (Taguchi/RSM) along with a suitable optimisation method has enhanced the machining performance of EDM process. However, the past studies focus on either Taguchi or RSM individually, with primary focus on optimisation. Some of the studies optimised MRR or SR independently, without addressing the conflicting nature of these objectives or using multi-objective approaches. The application of machine learning techniques such as Naive Bayes with an integrated approach of DOE technique for predictive modelling has not been explored in depth. The integrated approach among the statistical modelling and data-driven decision-making tools, such as entropy-based weighting, has limited applications of past studies.

Based on the studied literature, a comparative study of Taguchi and BBD-RSM methods has not yet been reported on simultaneously studying the effects of process variables on SR, and MRR in WEDM of titanium alloys. Thus, the current study focuses on the modelling of WEDM process at numerous input process environments using Taguchi and BBD-RSM design, which, to the best of our knowledge, has not been reported in prior studies.. The variable input factors of WEDM process include T_on_, current, T_off_, whereas the response measures of MRR, SR were taken. The performance and adequacy of Taguchi and RSM models were assessed by using ANOVA, and coefficient of determination (R^2^). The study further uses NB to classify and predict machining outcomes, which enhances the decision-making process in manufacturing. The integration of Naive Bayes (NB) classification, a machine learning technique, for predictive modelling based on the empirical data generated by both DOE methods is a unique contribution. Lastly, the study employed entropy-based weighting to handle multi-objective optimisation of MRR and SR, which added a novel decision-support layer to the analysis. Authors consider that the present study pertaining to the comparison of DOE techniques will be useful for users in different domains of various applications.

## Materials and methods

### Experimental plan

**Fig. 1:**
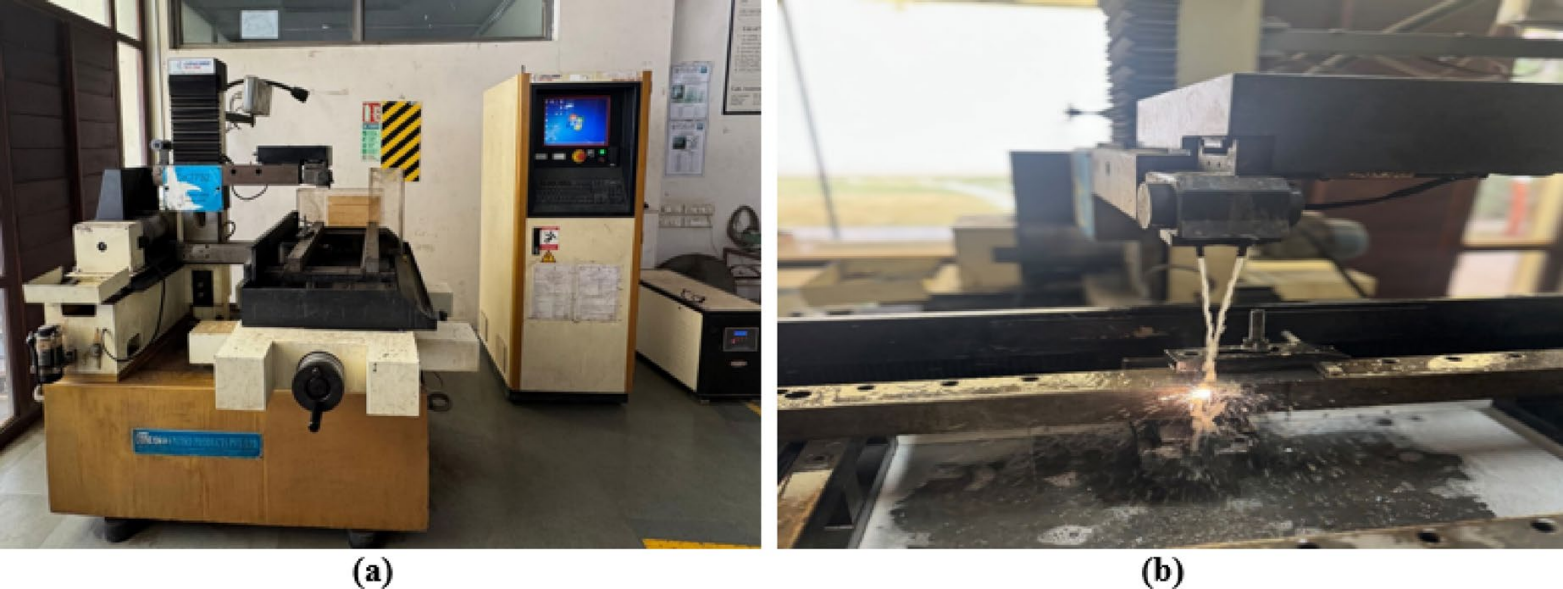
(**a**) Experimental setup, (**b**) enlarged view.

In the current study, a Concord-made WEDM setup was used to perform the experimental trials using EDM oil as dielectric fluid. Pure titanium was used as a work material, and a reusable molybdenum wire for the tool having a 0.18 mm diameter. Figure 1a,b shows the WEDM machining setup and an enlarged view of experimental trial, respectively. The variable input factors of EDM process include T_on_, I_p_, and T_off_, whereas MRR, SR were taken as the response measures. Table 1 shows the input factors with their levels. The input factors and their levels were selected on the basis of preliminary trials, literature survey, and machine limits. To understand the effect of multiple input factors, a strategic planning of experimentation is required. DOE techniques of Taguchi’s OA, and BBD matrix of RSM were used to design the experimental plan. For both DOE techniques, input factors and levels shown in Table 1 were utilised. Taguchi’s design needs fewer experimental trials, which in turn enhances the design efficacy^48^. Taguchi’s approach provides a linear interrelationship among the input factors and output measures^49^. BBD-RSM approach consists of a group of statistical and mathematical methods used for modelling and analysing the system performance by giving an interrelationship among the input factors and output measures^50^. It produces a non-linear second-order polynomial relationship^51^. All the experiments conducted by using both the DOE methods (Taguchi and BBD-RSM approach) were repeated thrice to confirm reproducibility and account for variability. The average values of the response variables (MRR and SR) were used in the analysis to enhance the statistical reliability and consistency of the results.

**Table 1:** Experimental conditions for DOE approaches.

Variables	Values
I_p_ (A)	3; 4; 5
T_on_ (µs)	60; 75; 90
T_off_ (µs)	6; 10; 14

The output measure, MRR, is referred to as the amount of material removal per unit time, and calculated by the weight of the work material measured per unit time. SR is critical to inspect the surface quality of the machined surfaces. Mitutoyo Surftest-410 was employed to measure SR of machined specimens by measuring at multiple locations.

### Naive Bayes classifiers (NB) algorithm

Machine learning has a wide range of applications in engineering problems, helping to improve performance, increase efficiency, and reduce costs^52^. These applications include predicting system behaviour and performance, detecting defects and anomalies, optimising processes, and quality control^53,54^. In particular, in civil, electrical, mechanical, and various industries, machine learning is used as a powerful tool to solve complex and innovative problems^55,56^. In machine learning, a Naive Bayes classifier consists of straightforward probability-based classifiers built upon Bayes’ theorem, assuming that random variables are independent^43^. The Naive Bayes algorithm is part of the family of generative learning algorithms. This means that it seeks to model the distribution of the inputs of a specific class or category. This approach works well for classifying various elements in the real world. The basis of the Naive Bayes algorithm is that the predicted variables in a machine-learning model are independent from each other. Event A conditional probability that event B occurs, written as $P\left(A|B\right)$, refers to the probability of event A given that event B has happened. Naive Bayes is built on the Bayes’ theorem used to estimate the conditional probabilities, which means nothing more than the occurrence probability of an event with some information about past events. Mathematically, Bayes’ theorem is expressed as Eq. (3):

3 $$P\left(A|B\right)=\frac{P\left(B|A\right)\times P(A)}{P(B)}$$

where, $P\left(A|B\right)$ represent the conditional probability of event A occurring given event B. In this study, the probability of each level of possibility against one another is equivalent to the ratio of A to B. This means that P(A) and P(B) are related to, in turn, the probabilities of events A and B.

A is recognised as the hypothesis, and B is the evidence, and consequently, P(A) and P(B) represent the prior probabilities of the hypothesis and evidence, respectively. $P\left(A|B\right)$ is referred to as the likelihood, and $P\left(B|A\right)$ indicates the conditional probability; therefore, Bayes’ theorem can be summarised as Eq. (4)^57^:

4 $$\text{contradiction}=\frac{\left(\text{likelihood}\right)\times (\text{probability of predicting a suggestion})}{\text{Probability of predicting an event}}$$

In the field of classification studies, Bayes’ theorem provides a formula for examining the membership of a class based on a set of input feature data and for calculating the probability of a data record. Suppose there are m classes including C_1_, C_2_, …, C_m_, and the probabilities of the classes P(C_1_), P(C_2_), …, P(C_m_) are available. Now, if the probabilities of the features x_1_, x_2_, x_3,…_ for each class are specified, Bayes’ theorem can be employed to calculate the probability that the available record belongs to class C_i_ (Eq. 5):

5 $$\begin{aligned} & P\left( {C_{i} |x_{1} ,x_{2} , \ldots ,x_{p} } \right) \\ & = \frac{{P\left( {x_{1} ,x_{2} , \ldots ,x_{p} |C_{i} } \right)P(C_{i} )}}{{P\left( {x_{1} ,x_{2} , \ldots ,x_{p} |C_{1} } \right)P\left( {C_{1} } \right) + P\left( {x_{1} ,x_{2} , \ldots ,x_{p} |C_{1} } \right)P\left( {C_{1} } \right) + \ldots + P\left( {x_{1} ,x_{2} , \ldots ,x_{p} |C_{m} } \right)P\left( {C_{m} } \right)}} \\ \end{aligned}$$

P (C_i_) is the prior probability of belonging to class C_i_ in the absence of other features. P (C_i_ |x_i_) is the likelihood of x_i_ belonging to class C_i_. To classify a record using Bayes’ theorem, the probability of its belonging to each class C_i_ is calculated. Then, the classification is performed based on the highest computed probability score using the above formula. Calculating P(x|C_i_) for a dataset with multiple features will require extensive computations. Therefore, a simplifying assumption is made that each input data point, given the class label in the sample, is independent from others. It is reasonable to assume that all predictor features in each class are independent from one another, allowing this equation to be simplified as follows (See Eq. 6):

6 $$P\left(x|{C}_{i}\right)=\prod_{k=1}^{n}P\left({x}_{k}|{C}_{i}\right)$$

**Fig. 2:**
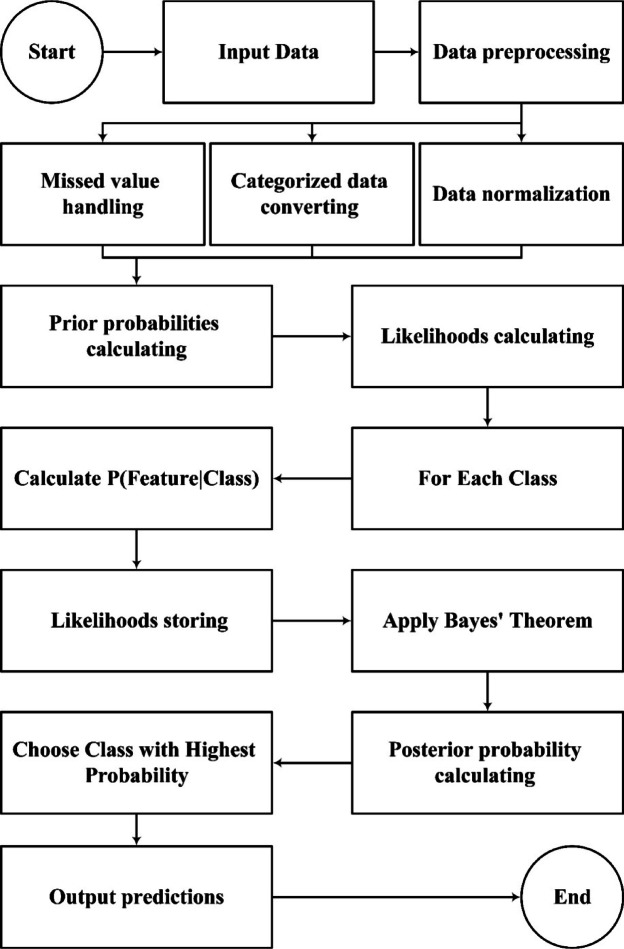
A view of NB steps algorithm.

A sample data point is represented by an n-dimensional vector, x = (x_1_, …, x_n_), which consists of samples from n features of class values A_1_, A_2_, …, and with the help of m classes C_1_, …, C_m_. In this case, P(x_k_|C_i_) = n/N, where n is the number of training samples of class C_i_ with value x_k_ for A_k_, and N is the total number of training samples belonging to C_i_^58^. A view of Naïve Bayes steps is presented in Fig. 2.

## Results and discussions

In the present research work, the aim is to investigate the variables of the WEDM process on the target factors. For this purpose, the design of the experiment with the aim of finding the effective practical tests has been discussed at the beginning. The results of the experimental tests have been examined by using DOE techniques of Taguchi, and BBD-RSM technique using the input factor conditions depicted in Table 2. Minitab v17 has been used to generate the single-order linear interrelationship among the input factors and output measures. Then, the superior empirical data have been selected assisted by the entropy weighting method. Finally, the prediction of the process findings has been measured by using the NB classifier. The statistical significance of the obtained finding, and empirical relations of DOE techniques were validated by using ANOVA, coefficient of determination (R^2^), and residual plots. The R^2^ values and residual analysis have proven to be effective as a cross-validation technique for the regression models^59,60^. The regression models facilitates the process optimization by accurately modeling the nonlinear interactions between input parameters and response variables^61,62^. For ANOVA, 95% confidence level was used to understand the impact of input factors on measures. This means the* P*-value of factor need not be more than 0.05 to have its impact for the elected response measure^63,64^.

**Table 2:** Design of experiments according to Taguchi and BBD-RSM techniques.

Test No	T_on_	T_off_	I_p_	MRR	SR
Design of experiments assisted by Taguchi					
1	60	6	3	1.2482	4.75
2	60	10	4	1.2968	4.03
3	60	14	5	1.1169	4.38
4	75	6	4	1.3678	5.64
5	75	10	5	1.2544	5.56
6	75	14	3	0.7646	4.44
7	90	6	5	1.4903	7.32
8	90	10	3	0.9965	6.49
9	90	14	4	0.9886	6.57
Design of experiments assisted by BBD-RSM					
1	60	14	4	0.9246	4.15
2	75	10	4	1.0737	5.31
3	90	10	3	0.9965	6.49
4	75	10	4	1.0645	5.17
5	90	6	4	1.3868	7.08
6	75	14	5	0.9985	6.17
7	90	14	4	0.9886	6.57
8	90	10	5	1.2389	7.13
9	75	10	4	1.0830	5.22
10	75	14	3	0.7646	4.44
11	75	6	5	1.5038	5.98
12	75	6	3	1.2802	5.87
13	60	10	5	1.4143	5.25
14	60	6	4	1.4771	4.89
15	60	10	3	1.0853	4.43

### Design of experiments

This section presents the experimental outcomes of the current study. As mentioned earlier, several variables affect the quality of the product obtained from the PMEDM process. In this research work, T_on_, T_off_, and I_p_ have been investigated as process variables. Various parameters can be considered as product quality indicators in this process, and in the current study, the effects of the above-mentioned variables on the two factors, namely MRR and SR, have been analyzed simultaneously. Based on the limitations of the setup, the operational feasibility of the WEDM process and also by utilising previous relevant research, the range of changes of the input variables has been defined, and the number of levels each has been considered equal to 3. In the full factorial mode, the number of practical experiments will be considered to 27. There are various methods for conducting experimental design. In order to reduce the costs of empirical tests and the time of conducting the research, Box-Behnken Design and Taguchi techniques have been used in the present study to design the experiment. Accordingly, the number of practical experiments in BBD and Taguchi techniques was reduced to, in turn, 15 and 9. The design of experiments performed by these methods based on the range of variations and levels of process variables is given in Table 2.

As can be seen in Table 2, five similar tests were obtained between the two designs of experiment techniques, and accordingly, the number of independent practical experiments was considered to be 19. In the next section, the analysis of the results obtained from BBD-RSM approach is described in detail.

#### Taguchi’s design approach

Table 2 shows Taguchi’s OA with L9 design matrix along with the response measures of MRR and SR. Minitab v17 has been used to generate the single-order linear interrelationship among the input factors and output measures. Equations 7, 8 depicted the interrelationship for MRR and SR, respectively.

7 $$\text{MRR }=1.271-0.00207\cdot {\text{T}}_{\text{on}}-0.05151\cdot {\text{T}}_{\text{off}}+0.1421\cdot {\text{I}}_{\text{p}}$$

8 $$\text{SR}=0.639+0.08022\cdot {\text{T}}_{\text{on}}-0.0967\cdot {\text{T}}_{\text{off}}+0.263\cdot {\text{I}}_{\text{p}}$$

Table 3 shows the ANOVA findings of MRR and SR response measures for Taguchi. The results revealed that the full regression model, along with T_off_ and I_p_ input factors, significantly influenced the MRR response. Among these variables, MRR was found to be most strongly associated with T_off_. T_off_ contributed the most (63.47%), followed by I_p_ (30.18%), and T_on_ (1.44%). The R^2^ value of 95.10% indicates that the empirical model accounts for 95.10% of the variation in MRR, while only 4.09% of the total variation remains unexplained. The close agreement between the R^2^ and adjusted R^2^ values further supports the model’s reliability, confirming a strong correlation between the observed and predicted outcomes. For SR response measure, the regression model terms significantly influenced the developed model for the entire model. The results showed that the complete set of regression terms, particularly the input variables of T_on_ and T_off,_ had a notable impact on the SR. IP had the least influence, whereas T_on_ has shown maximum impact with 86.87% involvement, trailed by T_off_ with 8.96% involvement. The R^2^ value of 94.79% implies that the empirical model justifies 94.79% of the developed model was able to explain 94.79% of the variation in SR. The close agreement between the R^2^ and adjusted R^2^ values further confirmed a strong relationship between the observed and predicted responses^65^. The proximity of the values shows a robust and adequate relationship between them.

**Table 3:** ANOVA for MRR, and SR for Taguchi design.

Source	DF	Adj. SS	Adj. MS	F-Value	*P*-Value
For MRR response measure^a^					
Regression	3	0.3851592	0.127197	32.27	0.001
T_on_	1	0.005788	0.005788	1.47	0.279
T_off_	1	0.254700	0.254700	64.83	0.000
I_p_	1	0.121104	0.121104	30.82	0.003
Error	5	0.019644	0.003929		
Total	8	0.401236			
For SR response measure^b^					
Regression	3	10.0012	3.3337	30.34	0.001
T_on_	1	8.6881	8.6881	79.07	0.000
T_off_	1	0.8971	0.8971	8.16	0.036
I_p_	1	0.4161	0.4161	3.79	0.109
Error	5	0.5494	0.1099		
Total	8	10.5506			

^a^R^2^ = 95.10%, R^2^ adj. = 92.17%, R^2^ pred. = 87.73%

^b^R^2^ = 94.79%, R^2^ adj. = 91.67%, R^2^ pred. = 78.56%

#### BBD-RSM technique

Table 2 depicts the BBD design matrix of RSM approach with MRR and SR values. SR. Equations 9, 10 depicted the generated second-order polynomial interrelationship among the input factors and output measures for MRR, and SR, respectively.

9 $$\begin{aligned} {\text{MRR }} = & 4.117 - 0.0634 \cdot {\text{T}}_{{{\text{on}}}} - 0.1533 \cdot {\text{T}}_{{{\text{off}}}} + 0.1286 \cdot {\text{I}}_{{\text{p}}} + 0.00036 \cdot {\text{T}}_{{{\text{on}}}} \cdot {\text{T}}_{{{\text{on}}}} \\ & + 0.00217 \cdot {\text{T}}_{{{\text{off}}}} \cdot {\text{T}}_{{{\text{off}}}} + 0.00064 \cdot {\text{T}}_{{{\text{on}}}} \cdot {\text{T}}_{{{\text{off}}}} \\ \end{aligned}$$

10 $$\begin{aligned} {\text{SR}} = & 16.18 - 0.1454 \cdot {\text{T}}_{{{\text{on}}}} - 0.627 \cdot {\text{T}}_{{{\text{off}}}} - 2.734 \cdot {\text{I}}_{{\text{p}}} + 0.001444 \cdot {\text{T}}_{{{\text{on}}}} \cdot {\text{T}}_{{{\text{on}}}} \\ & + 0.00718 \cdot {\text{T}}_{{{\text{off}}}} \cdot {\text{T}}_{{{\text{off}}}} + 0.2666 \cdot {\text{I}}_{{\text{p}}} \cdot {\text{I}}_{{\text{p}}} + 0.1014 \cdot {\text{T}}_{{{\text{off}}}} \cdot {\text{I}}_{{\text{p}}} \\ \end{aligned}$$

Tables 4 and 5 show the ANOVA findings of MRR and SR response measures, respectively, for RSM design. Statistical analysis using ANOVA was conducted on the response values with the results for MRR as shown in Table 4. The model F-value of 63.39 through a* P*-value of 0.000 confirmed the model’s significance. The linear model terms, consisting of all WEDM process variables, had a significant impact on the response. The highest F-value of 278.89 for T_off_ shows that it is the most influential factor affecting MRR, followed by current. The square model terms were also significant, with T_on_ × T_on_ having a strong impact. The other square and two-way interaction terms were found to be non-significant due to their greater* P*-values. The two-way interaction term T_on_ × T_off_ indicated that the interaction effect was not statistically significant. Similarly, the square interaction T_off_ × T_off_ also showed a* p*-value of 0.147, suggesting insignificant impact on the response variable. The lack-of-fit value was recorded as 0.086, indicating that the model term fit the data. The insignificance of lack-of-fit suggests the adequacy of model^37^. The R^2^ value of 97.94% and adjusted R^2^ of 96.40% indicate that the model is highly acceptable, explaining most of the variation in MRR with minimal error.

**Table 4:** ANOVA for MRR using BBD design of RSM.

Source	DF	Adj. SS	Adj. MS	F-Value	*P*-Value
Model	6	0.6626	0.1104	63.39	0.000
Linear	3	0.6287	0.20959	120.30	0.000
T_on_	1	0.0105	0. 0105	6.05	0.039
T_on_	1	0.4859	0.4859	278.89	0.000
I_p_	1	0.1323	0.1323	75.95	0.000
Square	2	0.0279	0.0139	8.02	0.012
T_on_ × T_on_	1	0.0248	0.0248	14.25	0.005
T_off_ × T_off_	1	0.0044	0.0044	2.57	0.147
2-way-interaction	1	0.0059	0.0059	3.42	0.102
T_on_ × T_off_	1	0.0059	0.0059	3.42	0.102
Error	8	0.0139	0.0017		
Lack-of-fit	6	0.0137	0.0022	26.89	0.086
Pure error	2	0.0001	0.0000		
Total	14	0.6766			

R^2^ = 97.94%, R^2^ Adj. = 96.40%, R^2^ pred. = 88.77%

**Table 5:** ANOVA for SR using BBD design of RSM.

Source	DF	Adj. SS	Adj. MS	F-Value	*P*-Value
Model	7	12.5416	1.79166	119.19	0.000
Linear	3	11.2577	3.75255	249.65	0.000
T_on_	1	9.1229	9.12286	606.92	0.000
T_on_	1	0.7719	0.77190	51.35	0.000
I_p_	1	0.13629	1.36290	90.67	0.000
Square	3	0.6263	0.20875	13.89	0.002
T_on_ × T_on_	1	0.3897	0.38970	25.93	0.001
T_off_ × T_off_	1	0.0487	0.04872	3.24	0.115
2-way-interaction	1	0.2625	0.26248	17.46	0.004
T_on_ × T_off_	1	0.6577	0.65772	43.76	0.000
Error	1	0.6577	0.65772	43.76	0.000
Lack-of-fit	7	0.1052	0.01503		
Pure error	5	0.0948	0.01897	3.66	0.229
Total	2	0.0104	0.00519		

R^2^ = 99.17%, R^2^ Adj. = 98.34%, R^2^ pred. = 95.95%

The ANOVA findings for SR response were summarised in Table 5. The entire model, including 2-way interactions, square interactions and all linear terms, significantly influenced the SR response. Among the linear terms, T_on_ exhibited the highest contribution, trailed by T_off_ and I_p_. Square interaction terms of T_on_ × T_on_, T_off_ × T_off_, Ip × Ip, and two-way interaction of T_off_ × I_p_ showed the significant effect. The T_on_ × T_off_ interaction had a* p*-value of 0.000, indicating a highly significant effect on SR. The square terms, T_on_ × T_on_, and I_p_ × I_p_ also showed significance, with* p*-values well below 0.05, highlighting nonlinear behaviour and interaction among the input parameters affecting SR. The lack-of-fit value was recorded as 0.229, which is greater than 0.05, indicating that the model fits well and is acceptable. The R^2^ value of 99.17%, adjusted R^2^ of 98.34% confirm the model’s acceptability with minimal variation**.** The statistical adequacy of the RSM models is strongly supported by high R^2^ values, non-significant lack-of-fit results, and well-behaved residual plots, confirming that the developed models are both robust and reliable for predicting machining performance in WEDM.

### Effect of EDM variables on response measures

**Fig. 3:**
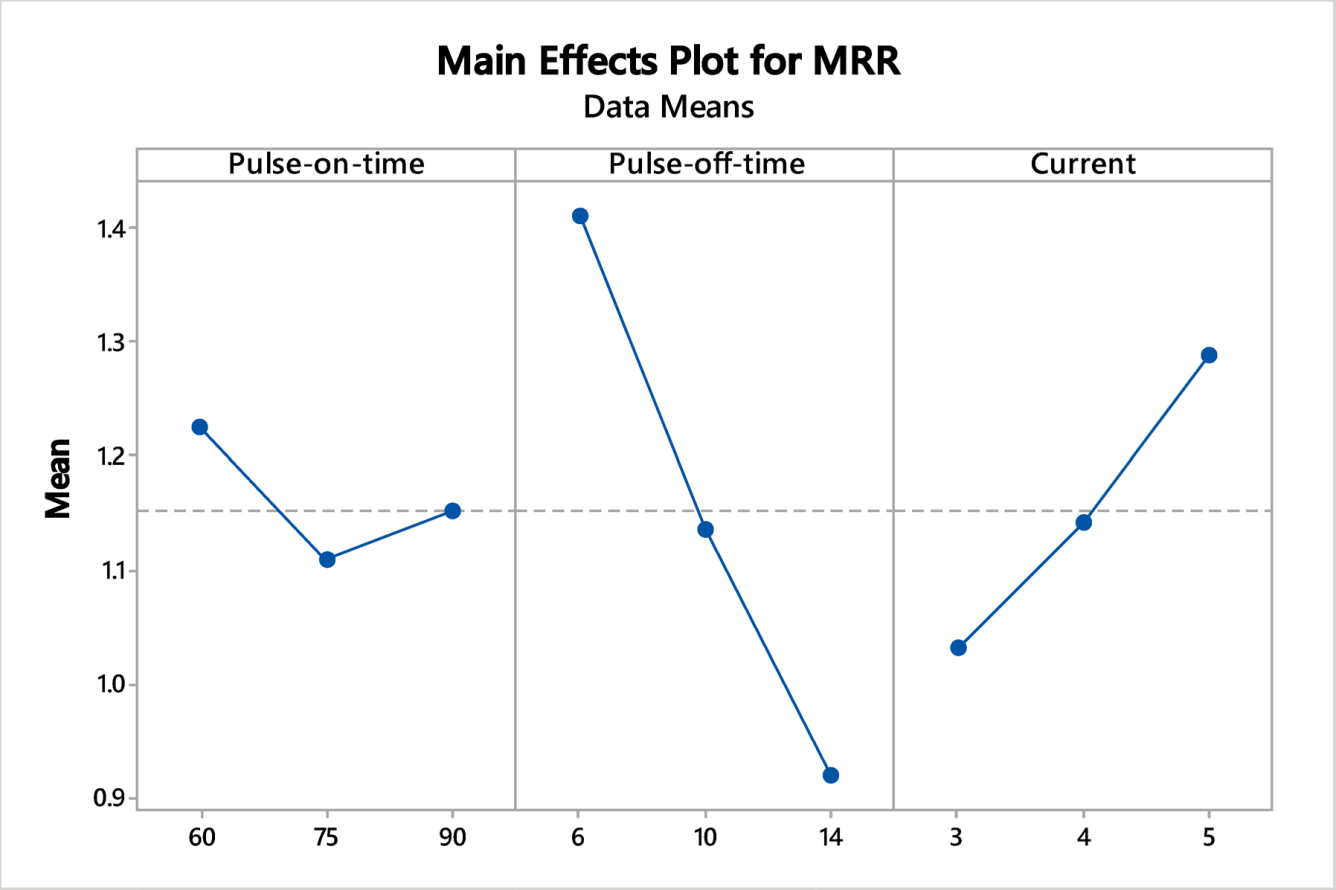
Influence of EDM factors on MRR.

**Fig. 4:**
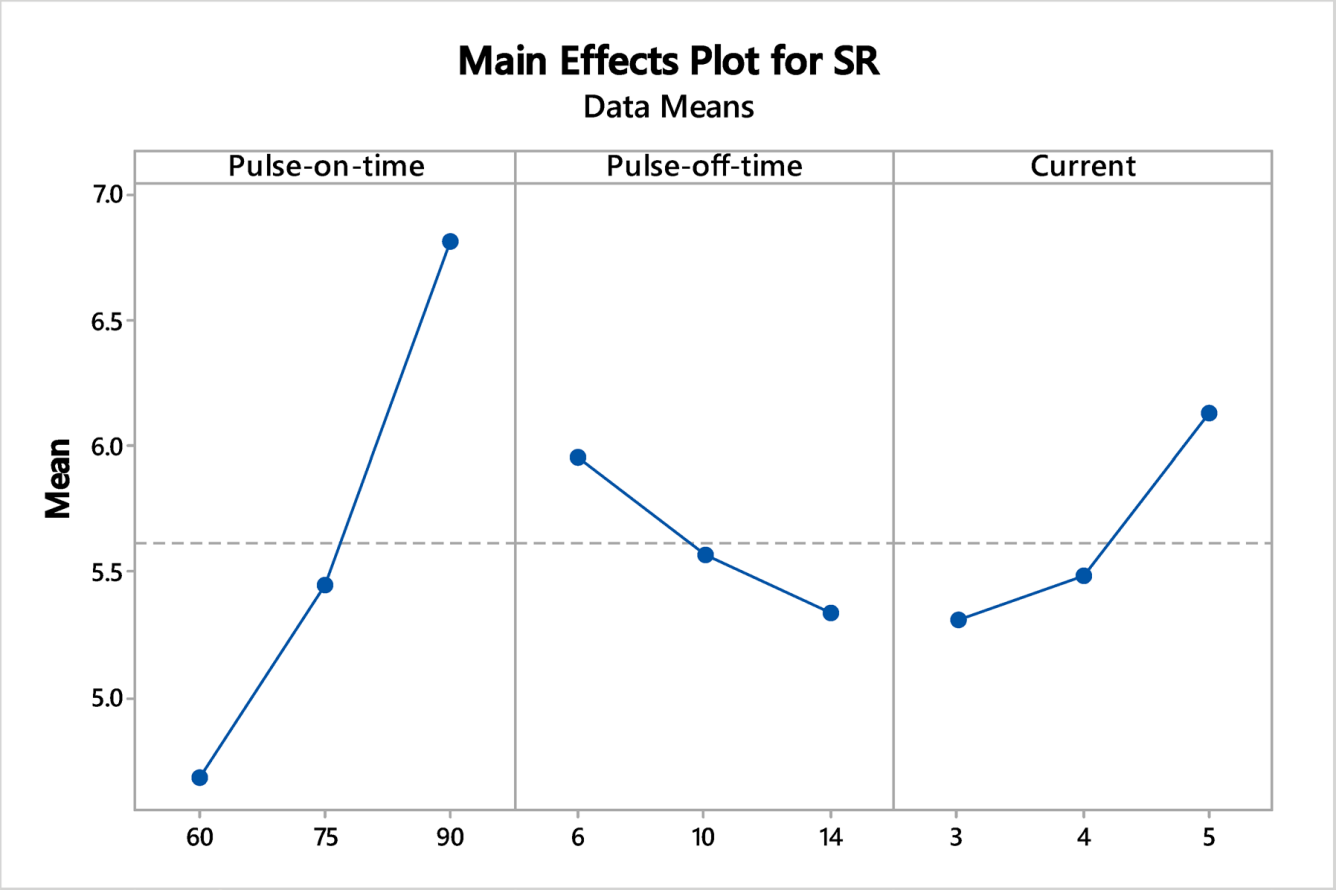
Influence of EDM factors on SR.

The effect of EDM factors (T_on_, T_off_, and I_p_) has been investigated on process indicators MRR and SR as shown in Figs. 3 and 4, respectively. The X-axis represents WEDM variables and the Y-axis represents the means of the SR corresponding to each factor. As the value of I_p_ increases, MRR also increases because it produces more discharge energy, causing more heat. As extra heat melts and vaporises material, it leads to more material removal^66^. Effect of T_off_ on MRR as rise in T_off_ results in decline of MRR as high T_off_ value induces less discharge energy^67^. Effects of T_on_ on MRR: As the input value of T_on_ increases leads to escalation in MRR because longer T_on_ means sparks last longer contributes to producing more heat^68^. Heat melts and vaporises more material, increasing erosion and improving MRR. As per these effect plots, higher MRR for the selected levels will be obtained at T_on_ at 60 µs, T_off_ at 6 µs, and I_p_ at 5 A.

Effect of WEDM parameters on SR output measurements was shown in Fig. 4. Effects of I_p_ on SR: As current increases, SR also increases because high current causes more discharge energy and heat to be generated. Intense heat leads to melting and vaporises the material, creating larger craters along the surface. Deep craters cause the surface to be less smooth and rougher^69,70^. Effect of T_off_ on SR: T_off_ increases cause a decrease in SR because longer T_off_ reduces spark intensity, which means less heat and smaller craters. Smaller creates cause smoother surface finish^71^. Effect of T_on_ on SR: Increase in T_on_ causes reduction in SR because at very high T_on_ values, SR increases slightly again. This is because high temperatures don’t allow enough time to flush away molten metal, leading to metal deposition on the surface^72^. As per these effect plots, higher MRR for the selected levels will be obtained at T_on_ at 60 µs, T_off_ at 14 µs, and I_p_ at 3 A. The main effect plots highlighted the necessity of optimize process parameters, as they reveal divergent input conditions for MRR and SR responses.

### *Analysis of practical findings *via* BBD-RSM approach using residual plots*

**Fig. 5:**
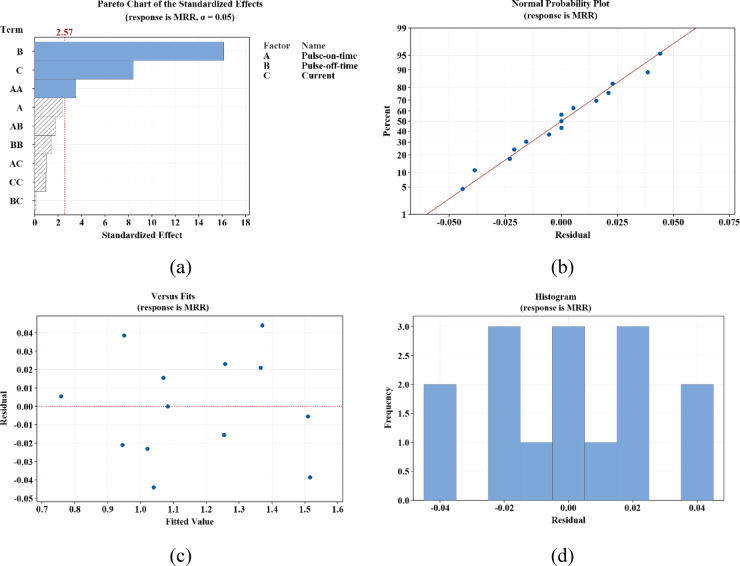
Regression analysis related to MRR.

**Fig. 6:**
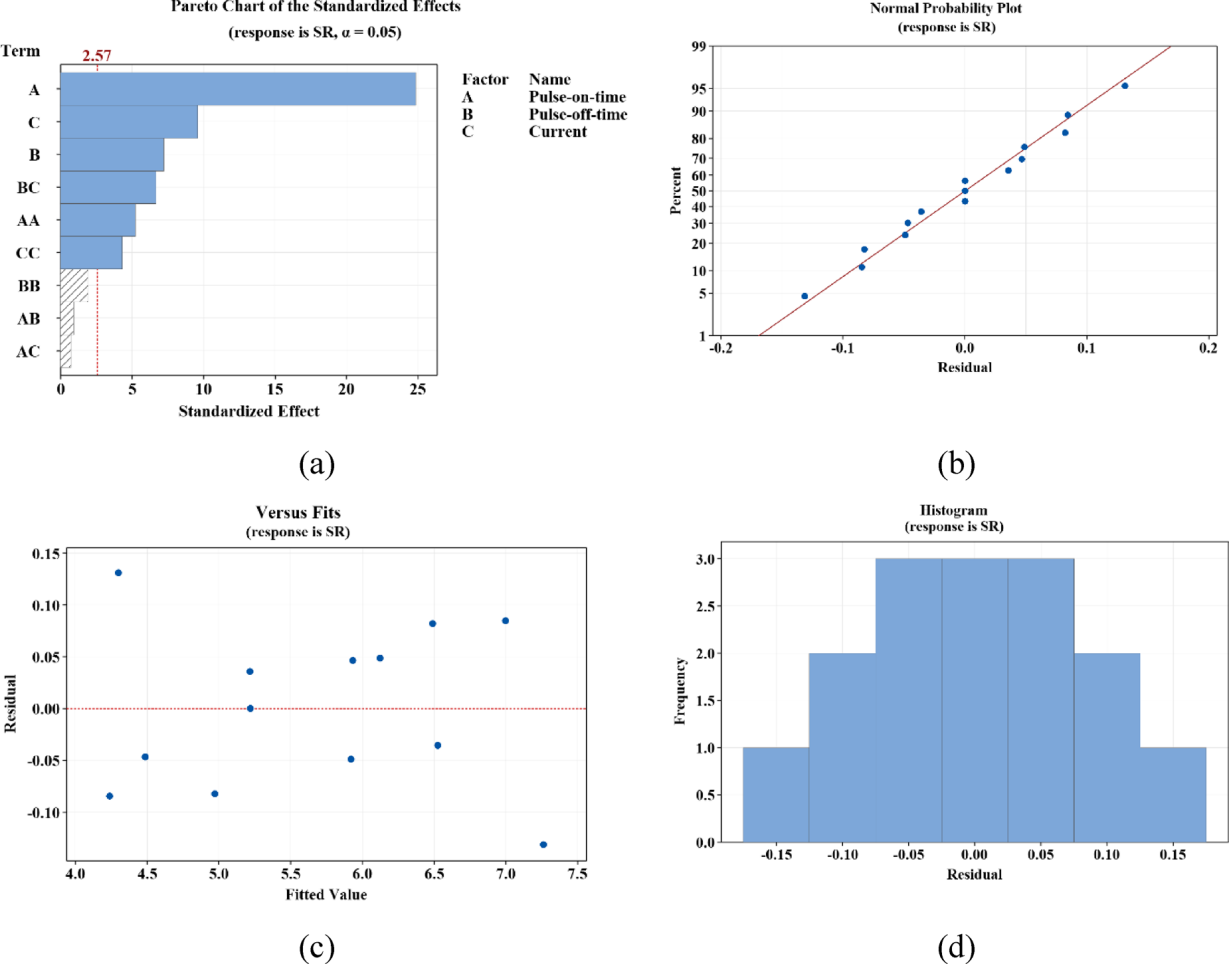
Regression analysis related to SR.

In this section, at first, the goal is to implement regression analysis to examine the influences of input variables on process indicators (MRR and SR) and the mathematical relationships governing them. Accordingly, the regression analysis outcomes for MRR and SR response variables are presented in Figs. 5 and 6, respectively.

As can be observed in these figures, the influential factors are highlighted in blue (see Figs. 5a and 6a). The normality of the residuals (the difference between the actual and predicted values) is demonstrated in Figs. 5b and 6b. The graph indicates the normality of the residuals. In section c, the graphs reveal the residual values versus the predicted values. In Figs. 5d and 6d, the frequency plots of the residuals are shown, confirming their normality. Based on the observations made in these two figures, it can be concluded that the machining variables and their range of changes are considered logical and effective^73^.

Table 6 shows the optimisation findings obtained from BBD-RSM. This analysis has been performed with Minitab software. As can be seen, for giving different weights to the response variables, the top three solutions are presented.

**Table 6:** The findings extracted from the BBD-RSM approach.

Solution	T_on_	T_off_	I_p_	SR Fit	MRR fit	Composite desirability
	SR Weight: 1			MRR Weight: 1		
1	60	6.00000	4.47475	5.05973	1.59062	0.540652
2	60	6.00000	4.89136	5.23723	1.66446	0.536105
3	60	6.64298	4.70169	5.09529	1.57489	0.534707
	SR Weight: 1			MRR Weight: 2		
1	60.0000	6.00000	5	5.29913	1.68495	0.489514
2	60.0000	6.00000	5	5.29913	1.68495	0.489514
3	60.3421	6.80912	5	5.26985	1.61054	0.470424
	SR Weight: 1			MRR Weight : 3		
1	60.0000	6.00000	5.00000	5.29913	1.68495	0.449308
2	60.0546	6.00000	4.63399	5.11752	1.61706	0.436399
3	60.1630	6.58196	5.00000	5.27520	1.63249	0.430380
	SR Weight: 2			MRR Weight: 1		
1	60	12.5455	3.16162	3.89179	0.91791	0.347896
2	60	13.4669	3.00000	3.72156	0.84466	0.347553
3	60	7.5408	4.26494	4.83037	1.42676	0.334641
	SR Weight : 3			MRR Weight: 1		
1	60	14.0000	3.00000	3.64888	0.81432	0.255947
2	60	11.9321	3.56286	4.10155	1.00617	0.241281
3	60	9.3356	3.60451	4.43678	1.19080	0.229368

Desirability value has been calculated for each of the tests presented in Table 1 (See Eqs. 11, 12). This value usually ranges between 0 and 1.

11 $$D_{i} = \frac{{Y_{i} - L_{i} }}{{U_{i} - L_{i} }}\,\,\,\,\,\,\,\,\,\,\,\,\,\,\,\left( {\text{for maximising}} \right)$$

12 $$D_{i} = \frac{{U_{i} - Y_{i} }}{{U_{i} - L_{i} }}\,\,\,\,\,\,\,\,\,\,\,\,\,\,\,\,\,\left( {\text{for minimising}} \right)$$

Here, Y_i_, L_i_ and U_i_ indicate, in turn, the actual value, the lower limit and the upper limit for criterion “i”. After calculating Desirability values for each criterion, the Composite Desirability can be computed. This is usually done using the geometric mean or by multiplying the Desirability values (See Eq. 13).

13 $$\text{D}={(\left({W}_{1}\times {D}_{1}\right)\times \left({W}_{1}\times {D}_{1}\right)\times \dots \times \left({W}_{n}\times {D}_{n}\right))}^\frac{1}{n}$$

In the above equation, D is the Composite Desirability value and n represents the number of criteria. It should be mentioned that W_i_ is the weight of the criterion “i” (See Table 7).

**Table 7:** The parameters in BBD-RSM for optimising.

Response	Goal	Lower (L_i_)	Target	Upper (U_i_)
SR	Minimizing	0	0	8
MRR	Maximizing	0	2	2

Based on the results of the two designs of experiments, it can be seen that all three variables and their ranges of variation affect the surface roughness and material removal rate indices. On the other hand, considering the same weight of the objective functions, no similar superior experiment was obtained through the two DOE. Accordingly, and in order to improve the accuracy of the Naive Bayes classifier, the set of experiments defined in these two design methods has been employed to train the NB algorithm for prediction of the process findings.

### Selecting the superior experiment based on entropy

The purpose of this section is to introduce the superior practical experiments. As mentioned in the previous section, in order to reduce the number of process experiments, the RSM-BBD approach was employed and based on it, 15 practical experiments were conducted. Another common technique in experimental design is the Taguchi method, and according to this method, the number of empirical tests is reduced to 9 (description and demonstration of the test conditions were presented in Section “Design of experiments”.). In order to improve the accuracy of training in the Naive Bayes algorithm, the total number of experiments (24 tests) proposed from the two aforementioned methods was used, some of which are repetitive.

**Fig. 7:**
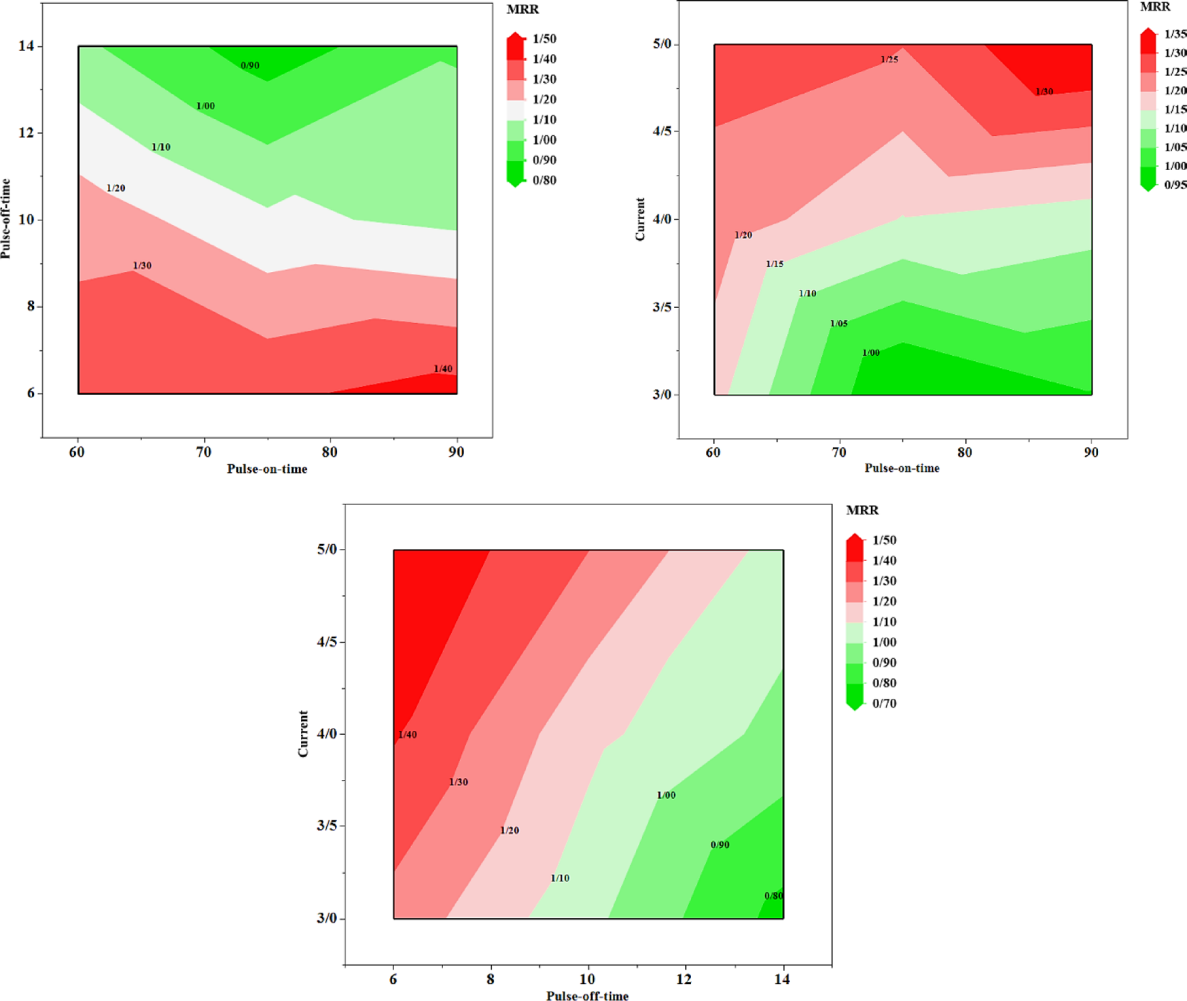
The contour plots corresponded to materials removal rate (MRR).

**Fig. 8:**
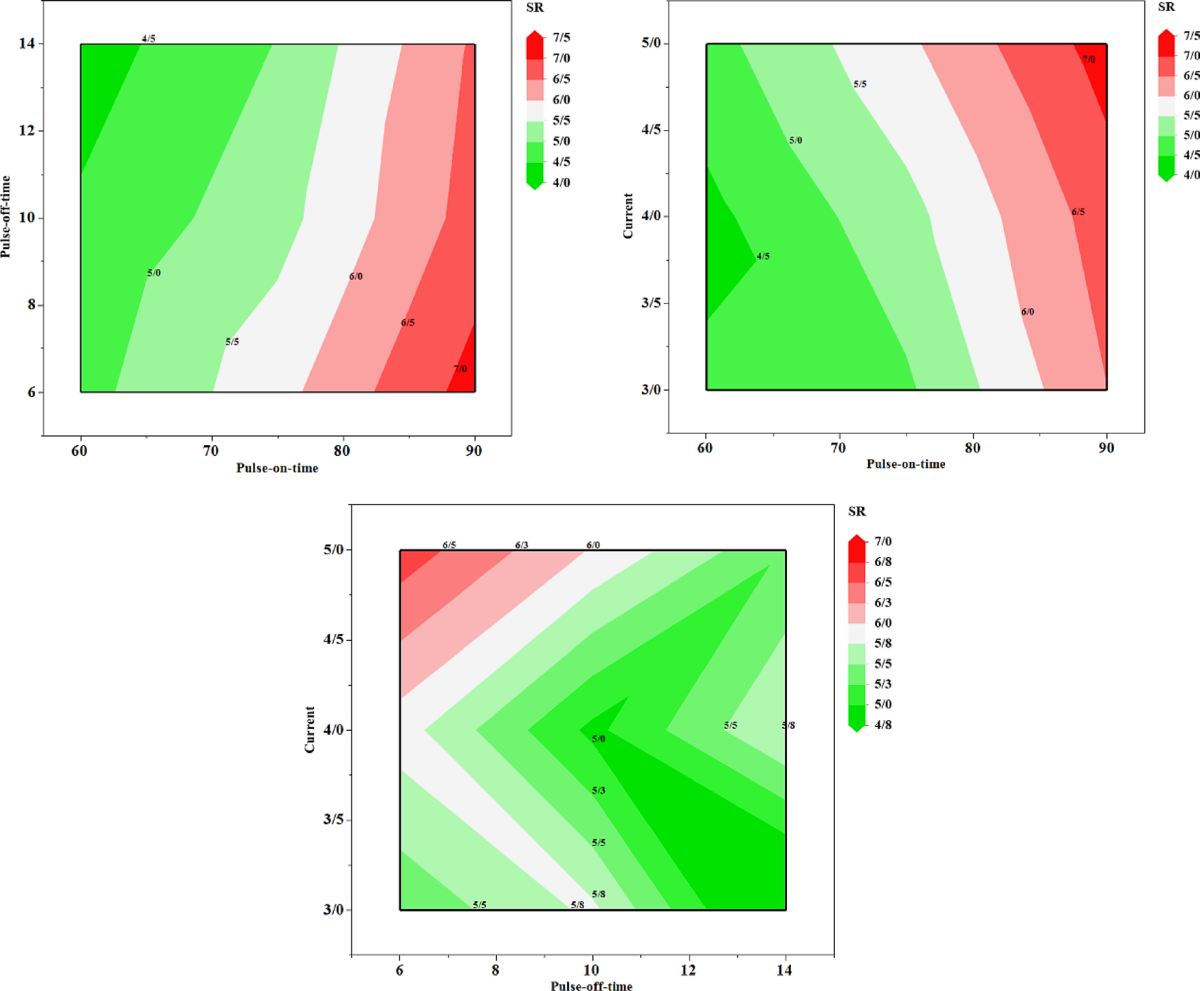
The contour plots corresponded to surface roughness (SR).

In Table 1, the results of the experimental tests are shown in discrete states. In order to demonstrate the continuous changes of MRR and SR indices, the contour plots of their variations based on the changes of the input variables are shown in Figs. 7 and 8, respectively. Based on the trend of changes observed in the contour plots, it can be concluded that the process variables and their range of changes are influential in the values of the two target factors.

Table 8 demonstrates the ranking results of 24 experimental tests. As it is shown, all experimental tests performed are listed in this table. Columns 1 to 3 of the table represent the characteristics of each alternative, and columns 4 and 5 indicate the decision-making criteria. Columns 6 and 7 show the linguistic values for the indicators. In columns 8 and 9, the normalised values of columns 4 and 5 are calculated. MRR indicator is a positive index, meaning that a higher value is more desirable, and the SR factor is a negative index, meaning that a lower value is more desirable. In the positive index, to normalise each option in the index, we divide it by the maximum value of the index, and the resulting number is between zero and one and the closer the number of each alternative to one, the more desirable it is. To normalise the negative index, the minimum index value is divided by the alternative values. The resulting number is between zero and one, and the closer it is to one, the more desirable it is. One of the most widely used methods for extracting the weight coefficients of response variables is the entropy technique, which was employed in the present research work (a comprehensive explanation of how the entropy method works is provided in Refs.^74,75^). Based on it, the weights coefficient of the MRR and SR response variables were obtained as 0.52 and 0.48, respectively. The score of each alternative is equal to the sum of the product of the index weight and the normalised value of the alternative in that index. In column 10 of Table 4, the score values of the different options are calculated and their rankings are shown in column 11. The top three alternatives are highlighted.

**Table 8:** Test rankings based on entropy criterion.

T_on_	T_off_	I_p_	MRR	SR	MRR_L_	SR_L_	MRR_N_	SR_N_	Score	Rank
60	6	3	1.2482	4.7500	Median	Low	0.83	0.8484211	0.8388554	5
***60***	***6***	***4***	***1.4771***	***4.8910***	***High***	***Low***	***0.9822485***	***0.8239624***	***0.9061572***	***2***
60	10	3	1.0853	4.4310	Median	Low	0.7217391	0.9095012	0.8120002	8
**60**	**10**	**4**	**1.2968**	**4.0300**	**High**	**Low**	**0.8623377**	**1**	**0.9285147**	**1**
*60*	*10*	*5*	*1.4143*	*5.2510*	*High*	*Median*	*0.9405099*	*0.7674729*	*0.8573275*	*3*
60	14	4	0.9246	4.1540	Low	Low	0.6148148	0.9701493	0.7856313	9
60	14	5	1.1169	4.3800	Median	Low	0.7427293	0.9200913	0.8279908	6
75	6	3	1.2802	5.8700	High	Median	0.8512821	0.6865417	0.772088	12
75	6	4	1.3678	5.6400	High	Median	0.909589	0.714539	0.8158245	7
75	6	5	1.5038	5.9800	High	Median	1	0.673913	0.8432434	4
75	10	4	1.0737	5.3120	Median	Median	0.7139785	0.7586596	0.7354576	16
75	10	4	1.0645	5.1700	High	Median	0.7078891	0.7794971	0.7423125	15
75	10	4	1.0830	5.2200	Median	Median	0.7201735	0.7720307	0.7451023	14
75	10	5	1.2544	5.5600	Median	Median	0.8341709	0.7248201	0.7816038	10
75	14	3	0.7646	4.4400	Low	Low	0.5084227	0.9076577	0.700343	17
75	14	3	0.7646	4.4400	Low	Low	0.5084227	0.9076577	0.700343	17
75	14	5	0.9985	6.1720	Low	Median	0.664	0.6529488	0.6586875	20
90	6	4	1.3868	7.0800	High	High	0.9222222	0.569209	0.7525216	13
90	6	5	1.4903	7.3200	High	High	0.9910448	0.5505464	0.7792883	11
90	10	3	0.9965	6.4900	Low	High	0.6626747	0.6209553	0.6426193	21
90	10	3	0.9965	6.4900	Low	High	0.6626747	0.6209553	0.6426193	21
90	10	5	1.2389	7.1300	Median	High	0.8238213	0.5652174	0.6995052	19
90	14	4	0.9886	6.5700	Low	High	0.6574257	0.6133942	0.6362589	23
90	14	4	0.9886	6.5700	Low	High	0.6574257	0.6133942	0.6362589	23

### Prediction of the findings assisted by Naive Bayes

**Fig. 9:**
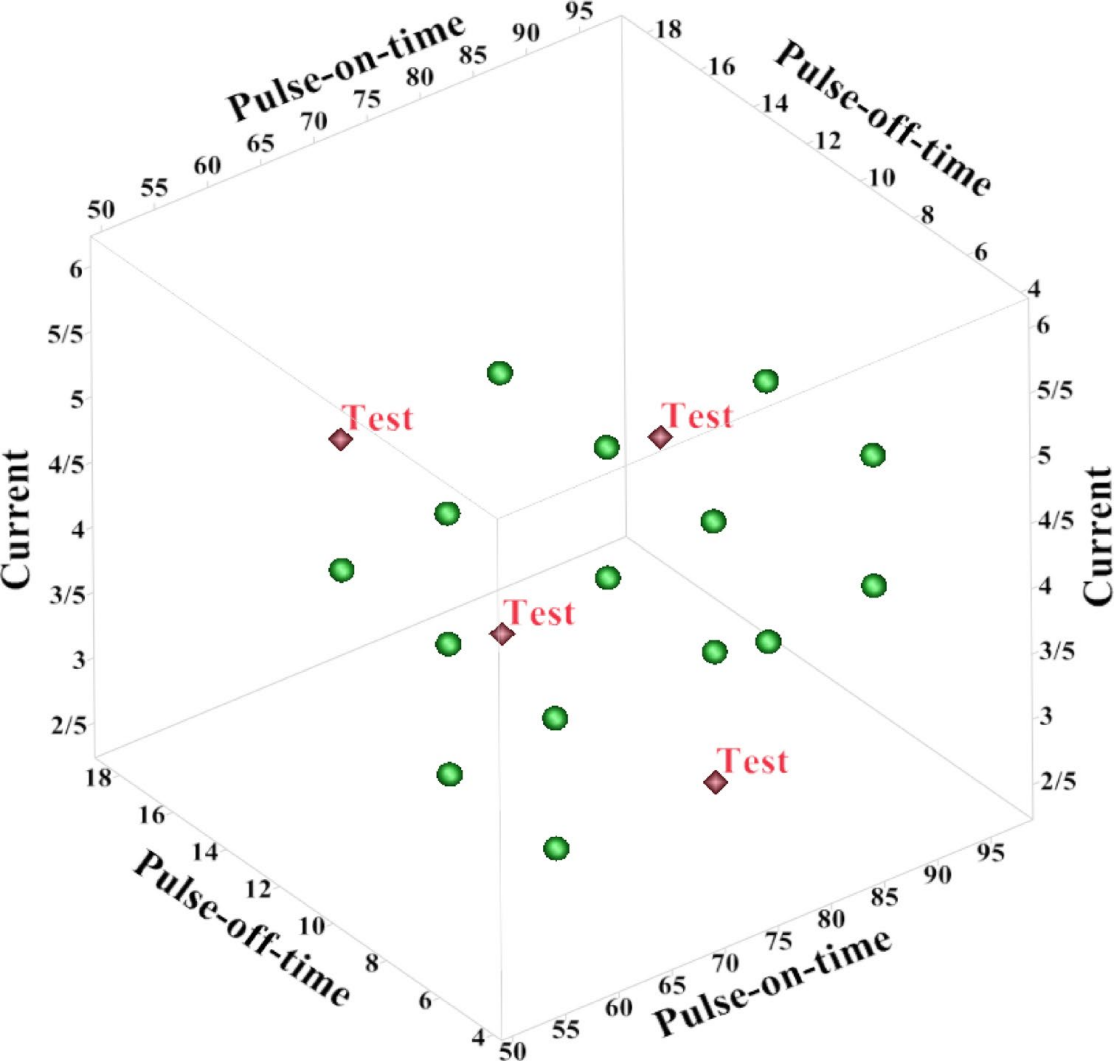
Display training and test data for changing process variables.

As explained earlier, Naive Bayes is one of the most popular probabilistic methods. In this classifier, the probability of a sample belonging to a specific class is estimated. Naive Bayes has an acceptable accuracy and is a fast technique for large data. From a total of 24 practical tests collected (obtained from BBD and Taguchi methods), repetitive data were removed and reduced to 19 tests. In this research, this data series is entered as input into the Naive Base method. Four of these practical experiments are considered as test data and the rest as training data. All data are shown in Fig. 9.

**Fig. 10:**
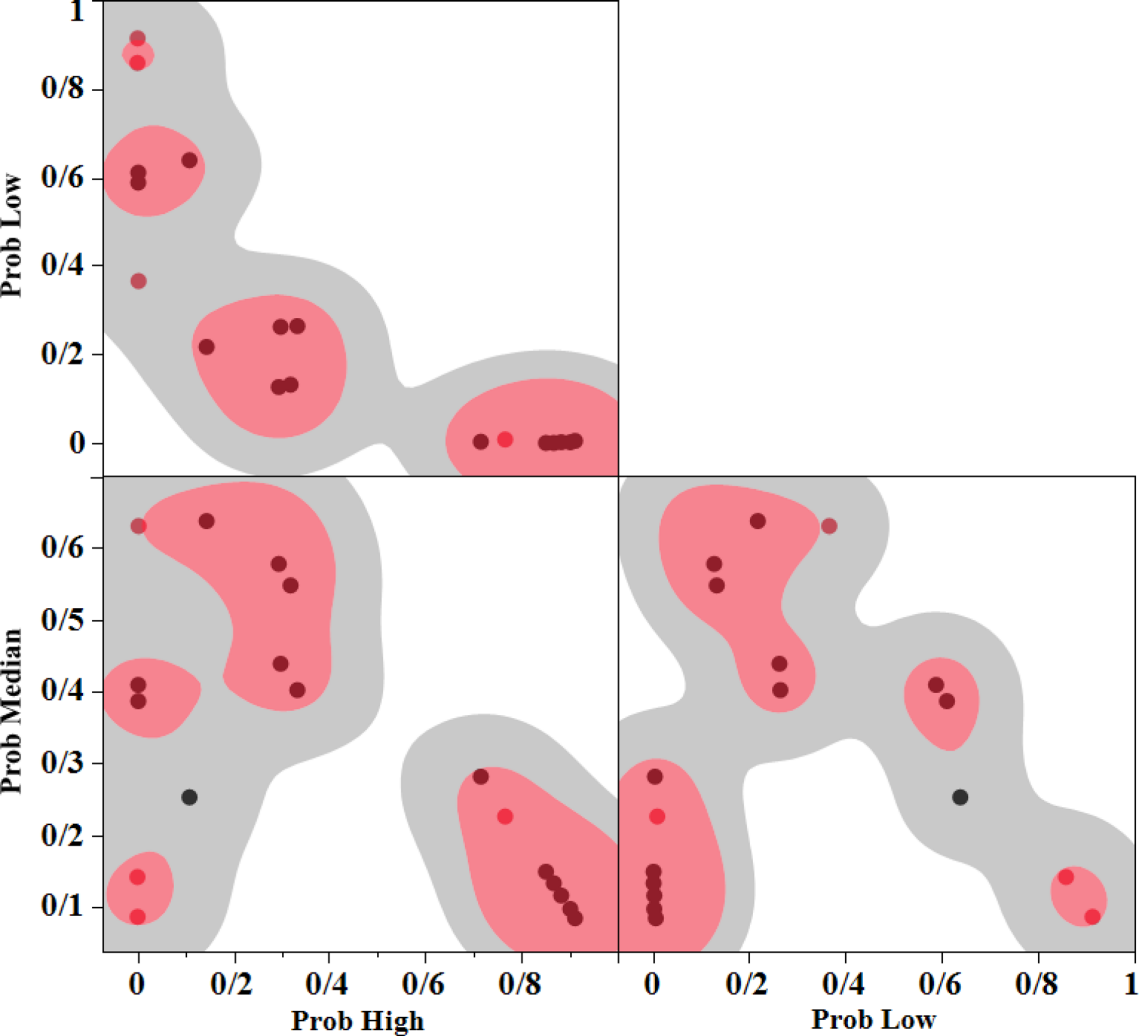
Scatterplot matrix for MRR.

For the MRR and SR response variables, three classes, namely High, Medium, and Low, are defined. Figure 10 shows the probabilities of the MRR data belonging to the three classes defined using the Naive Bayes method. Table 9 shows the value of MRR for test data. As can be seen, the prediction of data belonging to the defined classes works very well.

**Table 9:** NB results of test data (MRR).

Pulse-on-time	Pulse-off-time	Current	Prob high	Prob low	Prob median	MRR
60	14	5	0.002808123	0.3662362475	**0.6309556295**	Median
75	6	3	**0.7646928225**	0.0092236884	0.226083489	High
75	14	3	0.0022984914	**0.6106682138**	0.3870332947	Low
90	14	4	0.0008496325	**0.9130451624**	0.0861052051	Low

**Fig. 11:**
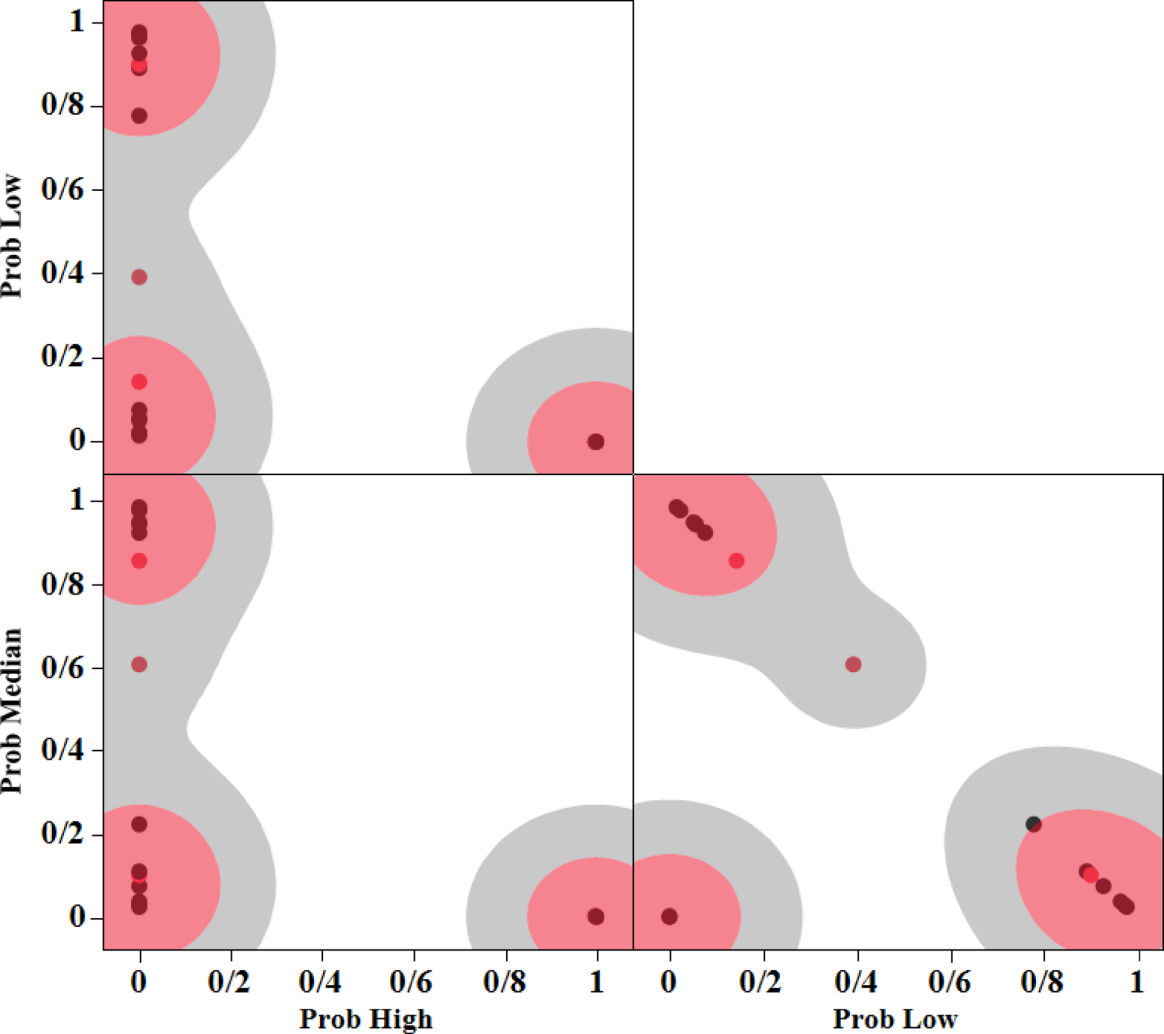
Scatterplot matrix for SR.

The matrix scatter plot provides a comprehensive visual tool to analyse the relationships between multiple variables in the context of Naive Bayes classification. In Figs. 10 and 11, in each of the three sections, the probability of each data point belonging to three categories (Low, Medium, and High) can be seen. In these plots, subclasses within the three main classes (Low, Medium, and High) are highlighted.

Figure 11 shows the probabilities of the surface roughness data belonging to the three classes defined using the Naive Bayes method. Table 10 demonstrates the value of surface roughness (SR) for test data series. As can be seen, the prediction of data belonging to the defined classes works very well.

**Table 10:** NB results of test data (SR).

Pulse-on-time	Pulse-off-time	Current	Prob high	Prob low	Prob median	SR
60	14	5	0	0.8980572793	0.1019427207	Low
75	6	3	0	0.1427661183	0.8572338817	Median
75	14	3	0	0.6081546252	0.3918453748	Low
90	14	4	0.9982608536	2.4477748e-6	0.0017366986	High

By comparing Tables No. 9 and No. 10 with Table 8, it can be realised that all four test data points are predicted correctly by the Naive Bayes model in both MRR and SR. Therefore, one can trust the accuracy of the Naive Bayes classifier in predicting the values of the response variables.

**Fig. 12:**
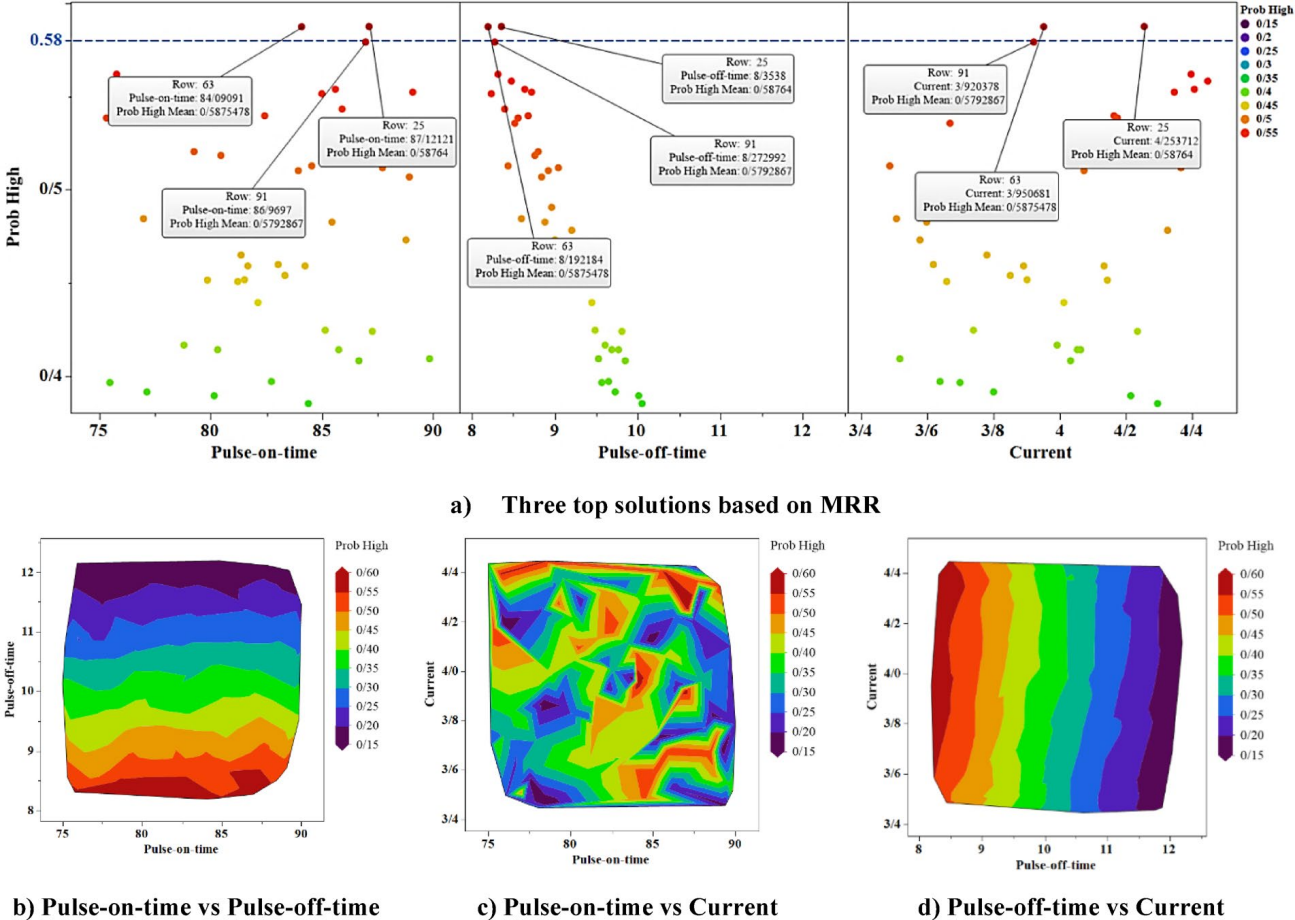
The findings for MRR simulation.

**Fig. 13:**
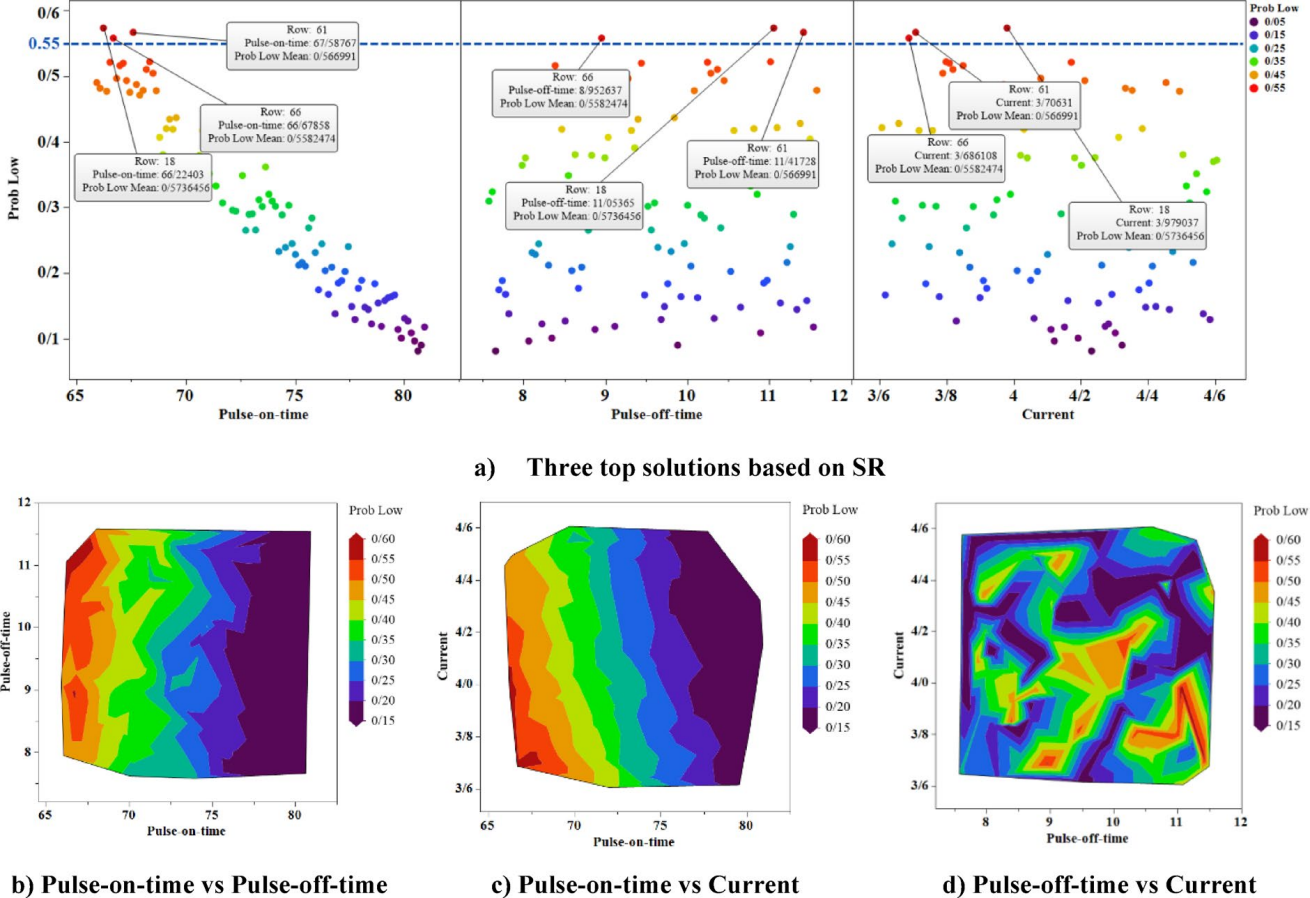
The findings for SR simulation.

Using the prediction model obtained from the Naive Bayes method, 100 random input data were given to the model, and the prediction results are shown in Figs. 12 and 13. Three input factor values have been generated using uniform random variables. For 100 randomly generated inputs, MRR and SR values have been calculated using the trained Naive Bayes model. At the top of Figs. 12 and 13, the probability of each of the 100 random data points belonging to the High class (for MRR) and Low class (for SR) is presented. In Fig. 12, the three points that are above the line with value of 0.58 are the points for which the probability of belonging to the High class is above 0.58. In Fig. 13, the three points that are above the line with value of 0.55 are the points for which the probability of belonging to the Low class is above 0.55. Since maximising the MRR value is of interest, the points with a higher probability of belonging to the High class are remarkable. Minimising the value of the SR response variable is the focus; therefore, the points with the highest probability of belonging to the Low class are important. In the lower part of Figs. 12 and 13, the probability contour plots for belonging to High class (for MRR) and Low class (for SR) have been drawn using 100 random data points. These charts illustrate how the probability of belonging changes with variations in the three input factors.

Furthermore, it is essential to validate the obtained predicted findings to confirm the accuracy and reliability of the developed models^76–78^. Thus, few validation experiments were conducted to confirm the optimal process parameters predicted by both the Taguchi and BBD-RSM models. The experiments were performed using the predicted optimal settings, and outcome responses (MRR and SR) were compared with the predicted values. The observed results of conducted trials have shown an acceptable deviation of less than 5% with the predicted values, thereby confirming the accuracy and reliability of the developed models. Additionally, the low residuals and close agreement among the predicted and actual values also show the model adequacy. These confirmation tests validate that the optimisation strategies used include entropy weighting and Naive Bayes prediction.

## Conclusion

In the present research work, a parametric study for enhancement of WEDM performance was carried out by analysing DOE techniques of Taguchi and BBD-RSM approach. T_on_, I_p_, and T_off_ were considered as machining variables and their effects on the MRR, and SR target factors were investigated, simultaneously. In order to predict the outcomes of the WEDM process, Naive Bayes classifier was employed. A summary of the research findings is given below:

- Based on the Taguchi and BBD-RSM approach, it can be observed that the selected variables and their range of changes had a significant influence on the quality of the machined part.
- The close agreement between the R^2^ and adjusted R^2^ values confirmed a strong relationship between the observed and predicted responses for both methods. The proximity of the values has shown a robust and adequate relationship between them.
- Regreesion analysis for MRR, and SR verified by using four residual plots has shown adequacy and acceptability of the models.
- Based on the entropy criterion, the weights of the SR and MRR factors were considered to 0.48 and 0.52, respectively. Regarding the obtained weights, the optimal values of the pulse-on-time, pulse-off-time and current variables were calculated to, in turn, 60 μs, 10 μs and 4 A. In this case, the values of the Sous and Moss indices were obtained as 2 and 3, respectively. In the optimal case, the values of the SR and MRR indices were obtained as 4.03 μm and 1.30 gr/sec, respectively.
- The test data series demonstrates the proper performance of Naive Bayes algorithm in predicting response variables.

## Data Availability

The datasets generated and analyzed during the current study are available from the corresponding author on reasonable request.

## References

[1] Malashin, I., Martysyuk, D. Powder particle classification with scanning electron microscopy images using machine learning techniques. Expert Syst. Appl. 128001 (2025).

[2] Lu, K. et al. High cycle fatigue limit prediction of machining foreign object damaged TC17 titanium specimen based on the theory of critical distances. *Sci. Rep.* **15**, 1–14 (2025). 40467723 10.1038/s41598-025-03449-y PMC12137747

[3] Li, J., Hu, J., Zhou, Q. & Zhang, Y. Transfer learning-based quality monitoring of laser powder bed fusion across materials. *Expert Syst. Appl.* **252**, 124150 (2024).

[4] Tang, J. et al. Tribological performance and lubrication mechanism of polyacrylamide as a high-efficiency water-based lubricant additive for titanium alloys. *Sci. Rep.* **15**, 1–15 (2025). 40461603 10.1038/s41598-025-00737-5 PMC12134339

[5] Ayeb, M., Turki, M., Frija, M. & Fathallah, R. Artificial neural network and ANFIS approaches for mechanical properties prediction and optimization of a turbine blade treated by laser shock peening. *Expert Syst. Appl.* **250**, 123911 (2024).

[6] Naji, F. A. A., Murtaza, Q. & Niranjan, M. Challenges and opportunities in nano finishing of titanium alloys for biomedical applications: A review. *Precis. Eng.* **88**, 81–99 (2024).

[7] Xu, W. et al. Experimental investigation on the properties and microstructure of hot isostatic pressed TA15 titanium alloy with hot-dip aluminum coating. *Sci. Rep.* **15**, 12744 (2025). 40222995 10.1038/s41598-025-97951-y PMC11994756

[8] El-Bassyouni, G. T., Mouneir, S. M. & El-Shamy, A. M. Advances in surface modifications of titanium and its alloys: implications for biomedical and pharmaceutical applications. *Multiscale Multidiscipl. Modeling, Exper. Design* **8**, 265 (2025).

[9] Liang, Y., Dai, C., Wang, J., Zhang, G., To, S., Zhao, Z. Typical applications and perspectives of machine learning for advanced precision machining: A comprehensive review. Expert Syst. Appl. 127770 (2025).

[10] Kang, S., Zhuo, X., Kong, L., Li, Y. & He, Y. Machining characteristics and process parameter optimization of Near-dry electrical discharge milling of titanium alloy. *Sci. Rep.* **15**, 8139 (2025). 40059190 10.1038/s41598-025-92830-y PMC11891323

[11] Selvarajan, L., Venkataramanan, K., Nair, A. & Srinivasan, V. Simultaneous multi-response Jaya optimization and Pareto front visualization in EDM drilling of MoSi2-SiC composites. *Expert Syst. Appl.* **230**, 120669 (2023).

[12] Kumar, S., Jayswal, S. C. Machining performance analysis of wire-EDM for machining of titanium alloy using multi-objective optimization and machine learning approach. *Proc. Institution Mechanic. Eng., Part E: J. Process Mech. Eng.* 09544089241288009 (2024).

[13] Sarker, B., Chakraborty, S., Čep, R. & Kalita, K. Development of optimized ensemble machine learning-based prediction models for wire electrical discharge machining processes. *Sci. Rep.* **14**, 23299 (2024). 39375462 10.1038/s41598-024-74291-x PMC11458828

[14] Korgal, A., Shettigar, A. K., Karanth, N. & Prabhakar, P. D. Advances in micro electro discharge machining of biomaterials: a review on processes, industrial applications, and current challenges. *Mach. Sci. Technol.* **28**, 215–265 (2024).

[15] Morovatdel, M., Osguei, A. T., Üstünel, Y. C. & Oliaei, S. N. B. Characterization of micro-wire electrical discharge machining surface texture by empirical mode decomposition. *Measurement* **242**, 116184 (2025).

[16] Guo, C. et al. Ultrasonic power adaptive adjustment for ultrasonic assisted die-sinking electrical discharge machining based on deep deterministic policy gradient. *Precis. Eng.* **94**, 808–819 (2025).

[17] Diviya, M. et al. Parametric investigation of W-EDM factors for machining AM60B conductive biomaterial. *Sci. Rep.* **14**, 216 (2024). 38168764 10.1038/s41598-023-50777-y PMC10761742

[18] Vincent, N. & John, F. R. A review of experimental investigations to attain productive and sustainable micro-electrical discharge machining process on metals and superalloys. *World J. Eng.* **22**, 1–13 (2025).

[19] Khoshaim, A. B., Muthuramalingam, T., Moustafa, E. B. & Elsheikh, A. Influences of tool electrodes on machinability of titanium α-β alloy with ISO energy pulse generator in EDM process. *Alex. Eng. J.* **63**, 465–474 (2023).

[20] Pendokhare, D., Chakraborty, S. A review on multi-objective optimization techniques of wire electrical discharge machining. Arch. Comput. Methods Eng. 1–43 (2024).

[21] Lamidi, S., Olalere, R., Yekinni, A., Adesina, K. Design of experiments (DOE): applications and benefits in quality control and assurance. (2024).

[22] Jankovic, A., Chaudhary, G. & Goia, F. Designing the design of experiments (DOE)–An investigation on the influence of different factorial designs on the characterization of complex systems. *Energy Build.* **250**, 111298 (2021).

[23] Zhang, H. H. et al. Optimization of high-speed channel for signal integrity with deep genetic algorithm. *IEEE Trans. Electromagn. Compat.* **64**, 1270–1274 (2022).

[24] Taguchi, G. Robust technology development. *Mech. Eng.-CIME* **115**, 60–63 (1993).

[25] Hou, T.-H., Su, C.-H. & Liu, W.-L. Parameters optimization of a nano-particle wet milling process using the Taguchi method, response surface method and genetic algorithm. *Powder Technol.* **173**, 153–162 (2007).

[26] Seidi, M., Yaghoubi, S. & Shirazi, A. Employment of fuzzy inference system in prediction of optimal solution area for thermal stress generated in the friction stir welding process. *Expert Syst. Appl.* **278**, 127342 (2025).

[27] Li, C., Xiao, Q., Tang, Y. & Li, L. A method integrating Taguchi, RSM and MOPSO to CNC machining parameters optimization for energy saving. *J. Clean. Prod.* **135**, 263–275 (2016).

[28] Jeyapaul, R., Shahabudeen, P. & Krishnaiah, K. Quality management research by considering multi-response problems in the Taguchi method–a review. *Int. J. Adv. Manuf. Technol.* **26**, 1331–1337 (2005).

[29] Box, G. E., Wilson, K. B. On the experimental attainment of optimum conditions, Breakthroughs in statistics: methodology and distribution, Springer, pp. 270–310 (1992).

[30] Susaimanickam, A., Manickam, P. & Joseph, A. A. A comprehensive review on RSM-coupled optimization techniques and its applications. *Arch. Comput. Methods Eng.* **30**, 4831–4853 (2023).

[31] Yaghoubi, S., Rabiei, F., Seidi, M. A comprehensive assessment on surface quality of machined wooden products via Box-Behnken design method. Wood Mater. Sci. Eng. 1–10 (2023).

[32] Lamidi, S., Olaleye, N., Bankole, Y., Obalola, A., Aribike, E., Adigun, I. Applications of response surface methodology (RSM) in product design, development, and process optimization. IntechOpen2022.

[33] Jagdale, M., Ambhore, N., Chaudhari, R., Kulkarni, A. & Abdullah, M. Experimental investigation of process parameters in Wire-EDM of Ti-6Al-4 V. *Sci. Rep.* **15**, 5652 (2025). 39955453 10.1038/s41598-025-90486-2 PMC11830067

[34] Hoang-Vuong, P., Huu-Phan, N., Shailesh, S. & Duc-Toan, N. Optimizing technological parameters in electrical discharge machining with graphene-coated aluminum electrodes for enhanced machining of titanium alloy: A Taguchi-TOPSIS approach. *Tribol. Ind.* **46**, 324 (2024).

[35] Singh, B. N. & Kirkup, S. M. Exploring MRR in wire EDM of titanium grade 5: A statistical analysis. *J. Mech. Eng. Adv.* **1**, 11–17 (2024).

[36] Chaudhari, R. et al. Experimental investigations of using MWCNTs and nano-graphene particles for the enhancement of machining performance using powder-mixed EDM of Udimet-720. *Int. J. Adv. Manuf. Technol.* **136**, 145–157 (2025).

[37] Selvarajan, L. et al. Performance analysis of EDM parameters on silicon nitride-titanium nitride composite for improving geometrical tolerances: An integrated GRA-RSM approach. *J. Chin. Inst. Eng.* **47**, 430–441 (2024).

[38] Kumar, P. M., Sivakumar, K. & Selvarajan, L. EDM machining effectiveness for Ti–6Al–4V alloy using Cu–TiB2 ceramic composite electrode: a parametric evaluation. *Ceram. Int.* **50**, 20118–20132 (2024).

[39] Mahanti, R. & Das, M. Sustainable EDM production of micro-textured die-surfaces: Modeling and optimizing the process using machine learning techniques. *Measurement* **242**, 115775 (2025).

[40] Chaudhari, R. et al. Implementation of passing vehicle search algorithm for optimization of WEDM process of nickel-based superalloy waspaloy. *Nanomaterials* **12**, 4394 (2022). 36558247 10.3390/nano12244394 PMC9781470

[41] Rajkumar, S., Selvarajan, L., Melese, K. G., Majora, M. & Wondimu, W. Optimization of electrical discharge machining parameters for enhanced performance on inconel 718 using Cu-Ni-B4C nanocomposite electrodes and advanced modeling techniques. *Mater. Res. Express* **11**, 095004 (2024).

[42] Kim, T. & Lee, J.-S. Exponential loss minimization for learning weighted naive bayes classifiers. *Ieee Access* **10**, 22724–22736 (2022).

[43] Romano, M., Contu, G., Mola, F. & Conversano, C. Threshold-based naïve bayes classifier. *Adv. Data Anal. Classif.* **18**, 325–361 (2024).

[44] Peretz, O., Koren, M. & Koren, O. Naive Bayes classifier–An ensemble procedure for recall and precision enrichment. *Eng. Appl. Artif. Intell.* **136**, 108972 (2024).

[45] Aggarwal, C. C. & Zhai, C. A survey of text classification algorithms. In *Mining Text Data* (eds Aggarwal, C. C. & Zhai, C.) 163–222 (Springer, 2012).

[46] Yu, S., Wang, Y., Chen, T., Li, M., Zhang, X., Huang, B., Xu, J., Wang, G. An inclined groove and its optimization design method for improving the energy performance at the saddle zone of axial flow pumps. Energy. 136527 (2025).

[47] Jhaveri, R. H., Revathi, A., Ramana, K., Raut, R. & Dhanaraj, R. K. A review on machine learning strategies for real-world engineering applications. *Mob. Inf. Syst.* **2022**, 1833507 (2022).

[48] Moayyedian, M., Qazani, M. R. C. & Pourmostaghimi, V. Optimized injection-molding process for thin-walled polypropylene part using genetic programming and interior point solver. *Int. J. Adv. Manuf. Technol.* **124**, 297–313 (2023).

[49] Aksoğan Korkmaz, A. & Toptaş, Y. Implementation of Taguchi method, ANOVA and regression analyses to enhance char yield by carbonization in lignite-biomass blended. *Int. J. Coal Prep. Util.* **45**, 264–280 (2025).

[50] Bheel, N. et al. Optimization of durability characteristics of engineered cementitious composites combined with titanium dioxide as a nanomaterial applying RSM modelling. *Sci. Rep.* **15**, 9428 (2025). 40108401 10.1038/s41598-025-94382-7 PMC11923098

[51] Sopandi, T. P. et al. RSM-optimized biochar production from young coconut waste (Cocos nucifera): Multivariate analysis of non-linear interactions between temperature, time, and activator concentration. *Ind. Crops Prod.* **223**, 120157 (2025).

[52] Tian, A., Zhang, W., Hei, J., Hua, Y., Liu, X., Wang, J., Gao, R. Resistance reduction method for building transmission and distribution systems based on an improved random forest model: A tee case study. *Build. Environ.* 113256 (2025).

[53] Zhang, H. H., Yao, H. M., Jiang, L. & Ng, M. Enhanced two-step deep-learning approach for electromagnetic-inverse-scattering problems: Frequency extrapolation and scatterer reconstruction. *IEEE Trans. Antennas Propag.* **71**, 1662–1672 (2022).

[54] Zhang, H. H. & Chen, R. S. Coherent processing and superresolution technique of multi-band radar data based on fast sparse Bayesian learning algorithm. *IEEE Trans. Antennas Propag.* **62**, 6217–6227 (2014).

[55] Sha, X., Zhu, Y., Sha, X., Guan, Z. & Wang, S. ZHPO-LightXBoost an integrated prediction model based on small samples for pesticide residues in crops. *Environ. Model. Softw.* **188**, 106440 (2025).

[56] Ma, C., Mu, R., Li, M., He, J., Hua, C., Wang, L., Liu, J., Totis, G., Yang, J., Liu, K. A multi-scale spatial–temporal interaction fusion network for digital twin-based thermal error compensation in precision machine tools. *Expert Syst. Appl.* 127812 (2025).

[57] Baumer, B. S. Kaplan, D. T., Horton, N. J. Modern data science with R, Chapman and Hall/CRC2017.

[58] Chapman, C., Feit, E. M. R for marketing research and analytics, Springer(2015).

[59] Selvarajan, L. et al. Spark eroding machining performance, surface textures and optimization strategies for ceramic composites: a review. *Arch. Civil Mech. Eng.* **25**, 87 (2025).

[60] Selvarajan, L., Venkataramanan, K., Devaraj, S. & Jesudas, T. The effect of rotary EDM parameters on the surface integrity and geometric parameters of holes in Si3N4-TiN electro conductive ceramics using DFA-RSM-ANN method. *Tribol. Int.* **200**, 110050 (2024).

[61] Wang, X., Su, H. & Liu, X. The impact of green technological innovation on industrial structural optimization under dual-carbon targets: The role of the moderating effect of carbon emission efficiency. *Sustainability* **17**, 6313 (2025).

[62] Sha, X., Si, X., Zhu, Y., Wang, S., Zhao, Y. Automatic three-dimensional reconstruction of transparent objects with multiple optimization strategies under limited constraints. *Image Vis. Comput.* 105580 (2025) .

[63] Wasif, M., Khan, Y. A., Zulqarnain, A. & Iqbal, S. A. Analysis and optimization of wire electro-discharge machining process parameters for the efficient cutting of aluminum 5454 alloy. *Alex. Eng. J.* **61**, 6191–6203 (2022).

[64] Vora, J. et al. Multi-response optimization and influence of expanded graphite on performance of WEDM process of Ti6Al4V. *J. Manuf. Mater. Proc.* **7**, 111 (2023).

[65] Chaudhari, R. et al. Multi-response optimization of WEDM process parameters for machining of superelastic nitinol shape-memory alloy using a heat-transfer search algorithm. *Materials* **12**, 1277 (2019). 31003478 10.3390/ma12081277 PMC6514827

[66] Chaudhari, R. et al. Pareto optimization of WEDM process parameters for machining a NiTi shape memory alloy using a combined approach of RSM and heat transfer search algorithm. *Adv. Manuf.* **9**, 64–80 (2021).

[67] Zadafiya, K., Kumari, S., Chatterjee, S. & Abhishek, K. Recent trends in non-traditional machining of shape memory alloys (SMAs): A review. *CIRP J. Manuf. Sci. Technol.* **32**, 217–227 (2021).

[68] Al-Amin, M., Abdul-Rani, A. M., Ahmed, R., Shahid, M. U., Zohura, F. T., Abd Rani, M. D. B. Multi-objective optimization of process variables for MWCNT-added electro-discharge machining of 316L steel. *Int. J. Adv. Manuf. Technol.* 1–20 (2021).

[69] Pour, M. & Ehsan Layegh, K. S. Influence of ZnO nanoparticle addition and spark peak current on EDM process of AISI 1045, AISI 4140, and AISI D3: MRR, surface roughness, and surface topography. *Int. J. Adv. Manuf. Technol.* **122**, 3703–3724 (2022).

[70] Chaudhari, R., Vora, J. J., Patel, V., López de Lacalle, L. & Parikh, D. Surface analysis of wire-electrical-discharge-machining-processed shape-memory alloys. *Materials* **13**, 530 (2020). 31979023 10.3390/ma13030530 PMC7040585

[71] Mohankumar, V. et al. A hybrid design of experiment approach in analyzing the electrical discharge machining influence on stir cast Al7075/B4C metal matrix composites. *Metals* **14**, 205 (2024).

[72] Markopoulos, A. P., Papazoglou, E.-L. & Karmiris-Obratański, P. Experimental study on the influence of machining conditions on the quality of electrical discharge machined surfaces of aluminum alloy Al5052. *Machines* **8**, 12 (2020).

[73] Yıldırım, Ö., Arıol, H., Sabah, E. Multiobjective optimization of dry batch micronized grinding of slaked lime in a vibrating mill via TOPSIS and ANOVA. *Separation Sci. Technol.* 1–14 (2025).

[74] Yaghoubi, S., Piccininni, A., Seidi, M. & Guglielmi, P. Multi-criteria optimization of the warm hydroforming process of an aluminum component based on the adaptive neuro-fuzzy inference system. *J. Manuf. Process.* **132**, 75–92 (2024).

[75] Yaghoubi, S., Seidi, M., Piccininni, A. Application of ANFIS–MADM hybrid technique to attain high-quality hydroformed component. *Proc. Instit. Mech. Eng., Part E: J. Process Mech. Eng.* 09544089251334416 (2024).

[76] Nair, A., Bizoń, W., Skoczypiec, S., Bogucki, R. & Selvarajan, L. Experimental exploration and optimization of dual-channel electrode micro-drilling in Inconel 617 using electrical discharge machining. *J. Braz. Soc. Mech. Sci. Eng.* **46**, 673 (2024).

[77] Dhanabal, P., Kalayarasan,M., Poovinan, A., Parthiban, M., Selvarajan, L. Optimized parameters of wire sparking process for machining of AlBe3 Alloy: a step toward enhanced machining performance. *J. Mater. Eng. Perf.*, 1–18 (2024).

[78] Manikandan, K., Thirugnanam, S., Selvarajan, L. & Senthilkumar, T. Study of correlation of machining performance and geometrical tolerances of Si3N4-TiN composites using EDM process. *SILICON* **16**, 3431–3451 (2024).

